# Mechanistic study of N-acetyltransferase 10 deficiency enhancing olaparib sensitivity in triple negative breast cancer by inhibiting RAD51 N4-acetylcytidine modification

**DOI:** 10.1016/j.isci.2025.112860

**Published:** 2025-06-09

**Authors:** Hui Li, Hao Wu, Siwei Li, Qin Wang, Guozheng Li, Xin Ma, Yajie Gong, Yijun Chu, Shengye Jin, Xi Chen, Xianyu Zhang, Da Pang

**Affiliations:** 1 Heilongjiang Clinical Research Center for Breast Cancer, Harbin Medical University Cancer Hospital, Harbin 150081, China; 2 Key Laboratory of Tumor Biotherapy of Heilongjiang Province, Harbin Medical University Cancer Hospital, Harbin 150081, China; 3 Department of Breast Surgery, Harbin Medical University Cancer Hospital, Harbin 150081, China; 4 Translational Medicine Research and Cooperation Center of Northern China, Heilongjiang Academy of Medical Sciences, Harbin 150081, China; 5 Department of Breast Surgery, The Second Affiliated Hospital of Harbin Medical University, Harbin 150086, China

**Keywords:** Molecular biology, Cell biology, Cancer

## Abstract

The treatment of triple-negative breast cancer (TNBC) is challenging due to the lack of common treatment targets, making standard hormonal and targeted therapies ineffective. While PARP inhibitors are promising for TNBC, they are only effective in homologous recombination (HR)-deficient cells with *BRCA1/2* mutations. Nevertheless, resistance to PARP inhibitors often develops. Thus, it is imperative to identify strategies or targets that can enhance the efficacy of PARP inhibitors. In this study, we demonstrated that TNBC cells lacking N-acetyltransferase 10 (NAT10) exhibited greater sensitivity to olaparib and extensive DNA double-strand breaks (DSBs). Mechanistically, NAT10 upregulates the N4-acetylcytidine (ac4C) modification of *RAD51* mRNA, enhancing its stability and increasing RAD51 expression. Remarkably, the combination of olaparib and remodelin, an inhibitor of NAT10, induced robust anti-tumor effects *in vitro* and *in vivo* by promoting DSBs. Our findings illuminate a potential therapeutic strategy targeting NAT10 to enhance olaparib efficacy in TNBC.

## Highlights

• NAT10 enhances RAD51 expression by stabilizing its mRNA through ac4C modification
• NAT10 inhibition increases olaparib sensitivity and DNA double-strand breaks in TNBC
• Co-treatment with remodelin and olaparib effectively suppresses TNBC progression

## Graphical abstract

**Figure:**
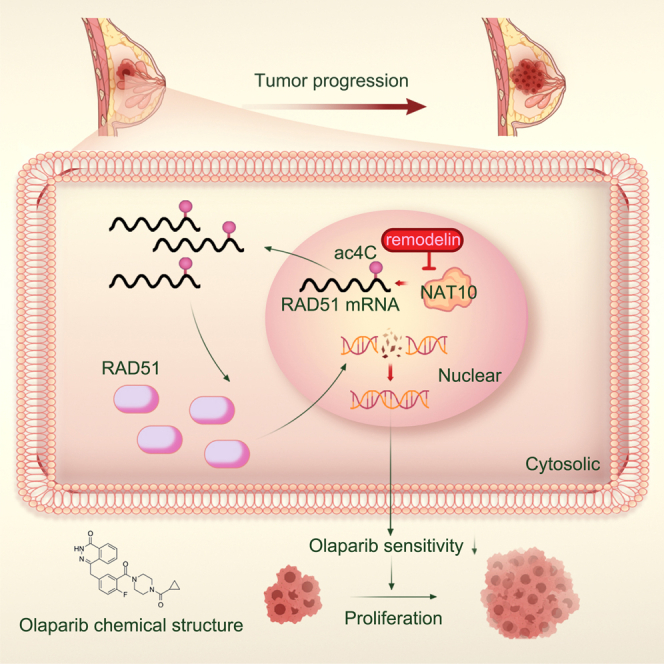

## Introduction

Breast cancer is the most frequent malignancy among women.^1^ The latest data from the American Cancer Society revealed that breast cancer accounts for 31% of all new cancer diagnoses among women in the United States.^1^ In addition, in the United States, it was estimated that there were 300,590 new cases of breast cancer and 43,700 breast cancer-related deaths in 2023.^1^ Triple-negative breast cancer (TNBC) accounts for approximately 15%–20% of all breast cancer cases.^2^^,^^3^^,^^4^ TNBC is known for its high aggressiveness within the spectrum of breast cancer, marked by high recurrence rates, a high incidence of distant metastases, and poor overall survival.^5^^,^^6^ The unique biological characteristics of TNBC, coupled with the lack of effective targeted therapies for estrogen receptor (ER), progesterone receptor (PR), or human epidermal growth factor receptor 2 (HER2), present significant challenges for its treatment.^2^^,^^7^^,^^8^ Therefore, there is an urgent need for extensive studies on targeted therapies to pave the way for effective treatment approaches for breast cancer.

Based on a study conducted by the Cancer Hospital of the Chinese Academy of Medical Sciences, 21.2% of 189 patients with TNBC had harmful *BRCA1/2* mutations, and 48.1% were defined as homologous recombination deficiency (HRD)-positive.^9^ HRD induces a vulnerability that can be utilized to selectively kill cancer cells.^10^ PARP inhibitors are the first small-molecule targeted drugs with synthetic lethal effects.^11^^,^^12^ PARP inhibitors block the catalytic activity of the PARP enzyme and induce PARP trapping, preventing the repair of DNA single-strand breaks and leading to DNA damage accumulation.^13^^,^^14^^,^^15^^,^^16^ Normal cells can repair DNA double-strand breaks (DSBs) through homologous recombination repair mechanisms, while tumor cells with BRCA deficiency can transform their DNA into lethal DSBs due to their inability to repair DSBs in a timely manner, leading to tumor cell death.^17^^,^^18^^,^^19^

PARP inhibitors, including olaparib and talazoparib, were approved for the treatment of HER2-negative advanced breast cancer with *BRCA1* and/or *BRCA2* genome mutations (*gBRCA1/2*).^20^^,^^21^ Although the clinical efficacy of PARP inhibitors as single drugs is promising, acquired drug resistance is a major problem in the treatment of breast cancer.^22^^,^^23^ After treatment with PARP inhibitors, breast cancer with *BRCA* mutation may obtain homologous recombination (HR) ability through various mechanisms, including BRCA-dependent/independent HR recovery.^15^ Therefore, there is an urgent need to identify molecular targets and potential strategies of combination therapy to expand the application of PARP inhibitors. So far, there are no direct inhibitors specifically targeting proteins that catalyze HR. Recent studies have focused on novel combination strategies using drugs that can induce HRD, finally leading to the indirect inhibition of HR and prompting the sensitivity to PARP inhibitors. For example, phosphoinositide 3-kinase (PI3K) inhibitors, histone deacetylase (HDAC) inhibitors, topoisomerase inhibitors, ataxia telangiectasia and Rad3-related protein (ATR) inhibitors, and checkpoint kinase 1 (CHK1) inhibitors affect homologous recombination repair through different mechanisms, resulting in DNA damage accumulation.^24^^,^^25^^,^^26^^,^^27^^,^^28^^,^^29^^,^^30^^,^^31^^,^^32^

As the only RNA N4-acetylcytidine (ac4C) modifying enzyme discovered so far, N-acetyltransferase 10 (NAT10) has been widely studied in both tumor and non-tumor diseases.^33^^,^^34^^,^^35^^,^^36^^,^^37^^,^^38^ Among them, the NAT10 writing protein is highly expressed in tumor tissues, catalyzing the acetylation of cytosine in various cancer-related RNAs to form ac4C modification and promoting the malignant behavior of tumor cells.^39^^,^^40^^,^^41^^,^^42^^,^^43^^,^^44^^,^^45^^,^^46^^,^^47^^,^^48^^,^^49^^,^^50^ Zhao et al. found that NAT10 is highly expressed in breast cancer and promotes the malignant behaviors of breast cancer cells. It facilitates the ac4C-modification of MDR1 and BCRP, which adversely affect the prognosis of breast cancer. Additionally, the inhibition of NAT10 sensitizes capecitabine-resistant breast cancer cells to chemotherapy both *in vitro* and *in vivo*.^50^ In terms of DNA damage repair, NAT10 plays a pivotal role by translocating from the nucleolus to the nucleoplasm through PARP1-mediated PARylation. In the nucleoplasm, NAT10 collaborates with the chromatin remodeling enzyme MORC2 to regulate DNA damage responses. It enhances the recruitment of DNA damage repair proteins by promoting the acetylation and stabilization of PARP1.^51^^,^^52^ Furthermore, the NAT10-mediated acetylation of MORC2 is essential for the activation of the G2 checkpoint following DNA damage, and its inhibition or depletion significantly increases cellular sensitivity to DNA-damaging agents.^49^ Genotoxic agents have been shown to upregulate NAT10 expression, enhancing cellular resistance to DNA damage.^53^

NAT10 also regulates mRNA stability and translation efficiency through ac4C modifications, which promote the expression of key nucleotide excision repair genes such as *DDB2*, thereby accelerating UVB-induced DNA damage repair.^54^ Notably, NAT10 enhances *AHNAK* mRNA stability and DNA damage repair by promoting ac4C modification, playing a crucial role in cisplatin-induced DNA damage and being closely associated with chemoresistance in bladder cancer.^45^ However, whether NAT10 can regulate DNA damage repair (DDR) in breast cancer by catalyzing the acetylation of RNA molecules remains to be further explored.

In this study, we found that NAT10 knockout enhanced the sensitivity of TNBC cells to olaparib *in vitro*, and increased olaparib-mediated cell apoptosis and DSBs. Mechanistically, NAT10 regulated the expression of RAD51 in an ac4C-dependent manner, thereby affecting HR repair. Moreover, the NAT10 inhibitor in combination with olaparib exerted a synergistic anti-tumor effect by accelerating the accumulation of DSBs *in vivo* and *in vitro*. Therefore, our findings underscore the important role of NAT10 in regulating HR and demonstrate the efficacy of NAT10 inhibitors in overcoming the resistance to olaparib for the treatment of TNBC.

## Results

### N-acetyltransferase 10 knockout sensitized triple-negative breast cancer cells to olaparib

Reports suggest that NAT10 plays a role in DNA damage, while olaparib exerts its effects through DNA damage-related mechanisms. Thus, we investigated the potential correlation between them.

Firstly, we found that the ac4C modification level remained unchanged after olaparib treatment in MDA-MB-231, BT-549, HCC1937, and MDA-MB-436 cells (Figure S1A).

Then, we constructed stable sh-NAT10 cell lines, including MDA-MB-231, BT-549, HCC1937, and MDA-MB-436 cells. qRT-PCR and Western blotting showed significant downregulation of NAT10 expression in sh-NAT10 cells compared to sh-NC cells (Figures S2A and S2B). We also constructed MDA-MB-231, BT-549, HCC1937, and MDA-MB-436 cell lines overexpressing NAT10 and validated them at protein and RNA levels (Figures S2C and S2D). As expected, silencing NAT10 reduced the overall ac4C levels of BC cells compared to control cells. NAT10 overexpression increased the ac4C levels of these cells compared to those of control cells (Figures S2E and S2F).

**Figure 1:**
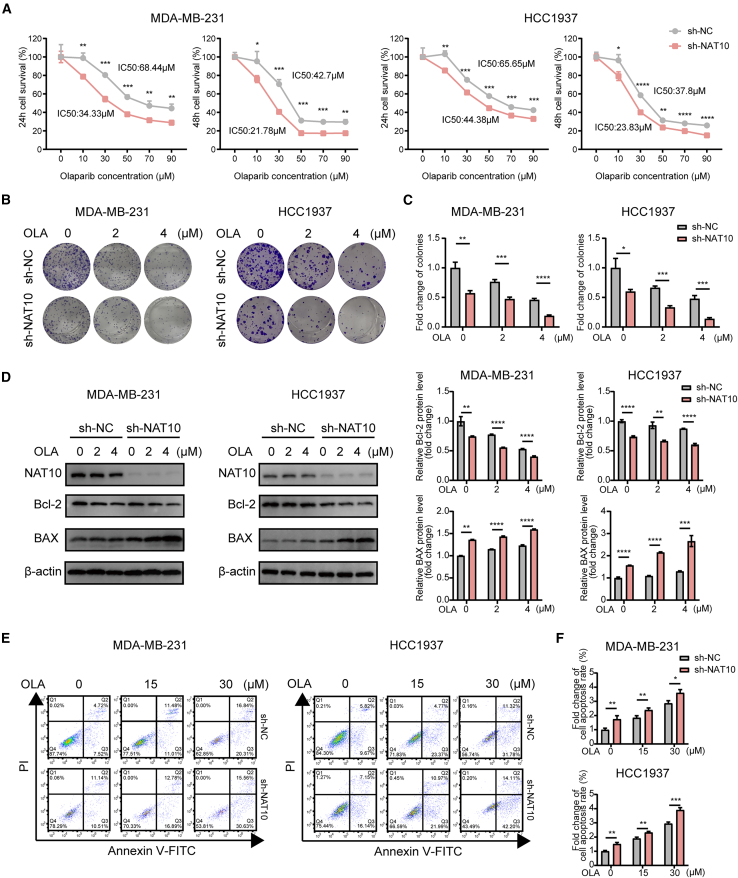
NAT10 knockout sensitized TNBC cells to olaparib (A) Cell survival analysis in MDA-MB-231 and HCC1937 cells with sh-NC or sh-NAT10 treated with olaparib for 24 h and 48 h. (B and C) Representative images (B) and quantification (C) of colony formation assay conducted on MDA-MB-231 and HCC1937 cells with sh-NC or sh-NAT10 after exposure to different concentrations of olaparib (0, 2, and 4 μM). (D) NAT10 knockout significantly enhanced the expression of the pro-apoptotic protein Bax and decreased the expression of the anti-apoptotic protein Bcl-2 in MDA-MB-231 and HCC1937 cells treated with olaparib. Western blotting was used to quantify protein levels across various concentrations of olaparib (0, 2, and 4 μM) in MDA-MB-231 and HCC1937 cell lines. (E and F) Flow cytometry analysis (E) and quantification (F) of apoptosis in MDA-MB-231 and HCC1937 cells with sh-NC or sh-NAT10 after treatment with different concentrations of olaparib (0, 15, and 30 μM). Data are presented as means ± SD. ∗*p* < 0.05, ∗∗*p* < 0.01, ∗∗∗*p* < 0.001, and ∗∗∗∗*p* < 0.0001. NAT10, N-Acetyltransferase 10; TNBC, triple-negative breast cancer.

Afterward, we used a stable sh-NAT10 cell line to explore the role of NAT10 in regulating the sensitivity of breast cancer cells to olaparib. Cell survival assays showed that NAT10 knockout sensitized TNBC cells to olaparib, since NAT10-deficient cells had a lower IC_50_ for olaparib (Figures 1A and S3A). The results were confirmed through colony-formation assays (Figures 1B, 1C, S3B, and S3C). Conversely, cell survival assays demonstrated that NAT10 overexpression reduced TNBC cell sensitivity to olaparib compared to NC cells (Figure S5A). Moreover, NAT10-overexpressing cells formed more colonies under olaparib treatment than NC cells (Figures S5B and S5C).

In addition, the increased expression of the pro-apoptotic factor Bax and decreased expression of the anti-apoptotic factor Bcl-2 were detected in sh-NAT10 TNBC cells treated with olaparib (Figures 1D and S3D). Flow cytometry showed that compared to the control group cells, sh-NAT10 cells treated with olaparib had a higher apoptosis rate (Figures 1E, 1F, S3E, and S3F). However, the decreased expression of Bax and increased expression of Bcl-2 were observed in NAT10-overexpressing TNBC cells treated with olaparib, accompanied by a lower apoptosis rate compared to control cells, as shown in Figures S5D–S5F.

Taken together, silencing NAT10 in TNBC cell lines increased their sensitivity to olaparib, overexpression of NAT10 had the opposite effect, suggesting that NAT10 inhibition can enhance the therapeutic efficacy of olaparib for treating TNBC.

### N-acetyltransferase 10 suppression enhanced olaparib-induced double-strand breaks

**Figure 2:**
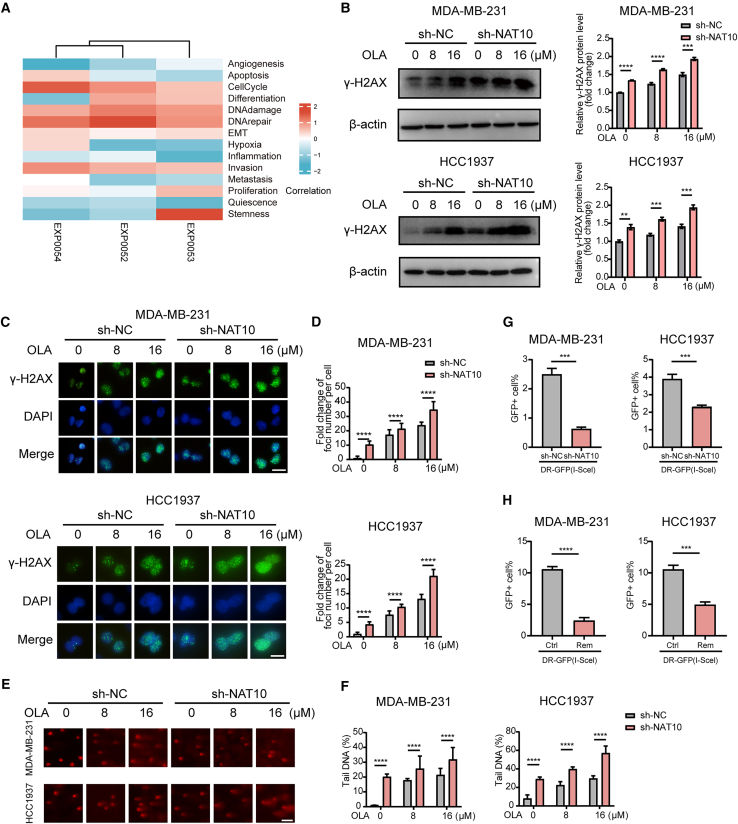
NAT10 suppression enhanced olaparib-induced DSBs (A) Heat maps of 14 cancer-related functional states in three breast cancer single-cell datasets. (B) Western blotting illustrating the levels of γ-H2AX, a marker of DNA damage, in MDA-MB-231 and HCC1937 cells with sh-NC or sh-NAT10 after treatment with different concentrations of olaparib (0, 8, and 16 μM). (C and D) Representative immunofluorescence images (C) and quantification (D) of γ-H2AX foci in MDA-MB-231 and HCC1937 cells with sh-NC or sh-NAT10 treated with increasing concentrations of olaparib (0, 8, and 16 μM). γ-H2AX foci are stained green, and nuclei are counterstained with DAPI (blue). Merged images highlighting the colocalization of γ-H2AX foci within the nuclei. Scale bar: 20 μm. (E and F) Comet assay images (E) and quantification (F) of tail DNA percentage in MDA-MB-231 and HCC1937 cells with sh-NC or sh-NAT10 after treatment with different concentrations of olaparib (0, 8, and 16 μM). Scale bar: 50 μm. (G) The frequency of GFP-positive (GFP+) cells of HR-mediated DSBs repair in MDA-MB-231 and HCC1937 cells with sh-NC or sh-NAT10. (H) The frequency of GFP+ cells of HR-mediated DSBs repair in MDA-MB-231 and HCC1937 cells treated with or without the NAT10 inhibitor remodelin. Data are presented as mean ± SD. ∗∗*p* < 0.01, ∗∗∗*p* < 0.001 and ∗∗∗∗*p* < 0.0001. NAT10, N-Acetyltransferase 10; DSBs, double-strand breaks; HR, homologous recombination.

We further elucidated the mechanisms by which NAT10 inhibition augmented the sensitivity to olaparib. The correlation analysis between genes of interest in different single-cell datasets and functional status in the CancerSEA: http://biocc.hrbmu.edu.cn/CancerSEA/ showed that *NAT10* may be involved in DNA repair in breast cancer, indicating that NAT10 inhibition may enhance the sensitivity to olaparib by regulating DNA damage (Figure 2A). It has been reported that in breast cancer, including TNBC, NAT10 plays a pivotal role in DNA damage repair by acetylating and PARylating proteins such as MORC2.^49^^,^^51^ To gain a deeper insight into the effect of NAT10 on DNA damage, we focused on γ-H2AX, a well-established marker of DNA double-strand breaks. Our data showed that NAT10 knockdown maintained higher levels of γ-H2AX in TNBC cells compared to control cells after treatment with different concentrations of olaparib (Figures 2B and S4A). Immunofluorescence data also indicated that olaparib dose-dependently increased the number of lesions marked by γ-H2AX in NAT10 knockout cells (Figures 2C, 2D, S4B, and S4C). To evaluate the degree of DNA damage, we conducted comet assays in different cell lines. In sh-NC cells, the percentage of comet tail DNA dose-dependently increased with olaparib treatment. Significantly, NAT10 knockout substantially increased the percentage of comet tail DNA in olaparib-treated cells (Figures 2E, 2F, S4D, and S4E). In contrast, NAT10 overexpression reduced γ-H2AX levels, decreased the number of olaparib-induced γ-H2AX foci, and significantly lowered the percentage of comet tail DNA in TNBC cells, indicating decreased DNA damage, as shown in Figures S5G–S5K.

As is well known, HR and NHEJ are the two primary pathways for DSB repair. Therefore, we attempted to explore the role of NAT10 in these processes. We used DR-GFP and EJ5-GFP reporter assays to assess the degree of HR and NHEJ, which are chromosomal reporter systems widely regarded as benchmark methods in the field of DNA repair. We found that NAT10 knockout and treatment with remodelin markedly inhibited HR efficiency in the breast cancer DR-GFP cell model (Figures 2G, 2H, S4F, and S4G) but not NHEJ efficiency in the EJ5-GFP cell model (Figures S6A and S6B).

Collectively, these results highlight the positive impact of NAT10 inhibition on olaparib-induced DSBs in TNBC, whereas NAT10 overexpression showed the opposite effect, reducing olaparib-induced DSBs and associated DNA damage.

### N-acetyltransferase 10 knockout sensitized triple-negative breast cancer to olaparib and promotes olaparib-induced double-strand breaks *in vivo*

**Figure 3:**
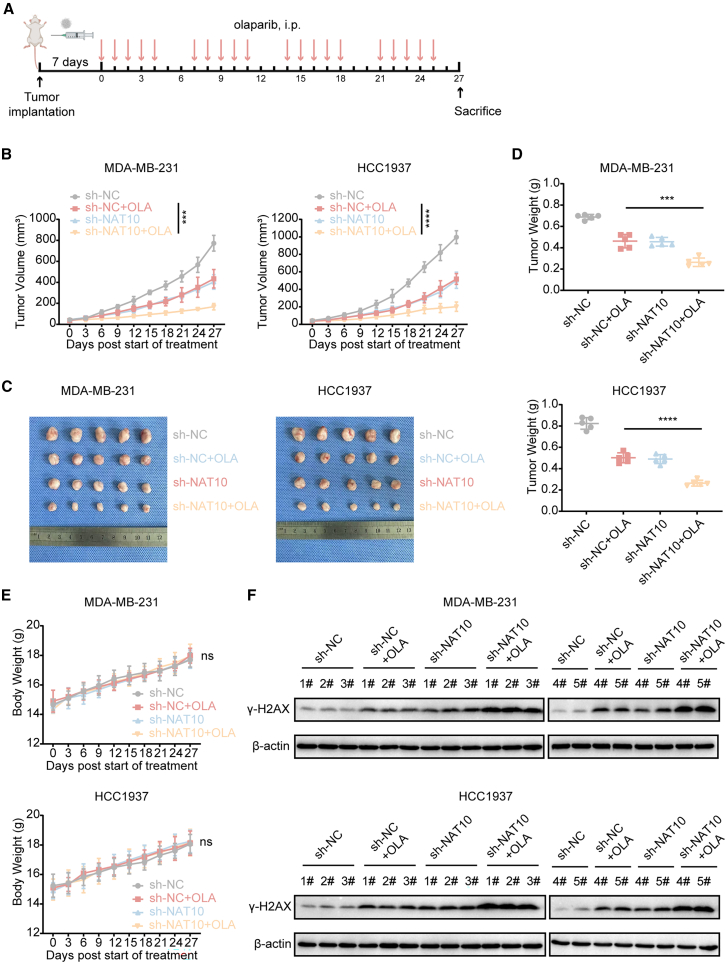
NAT10 knockout sensitized TNBC to olaparib and promotes olaparib-induced DSBs *in vivo* (A) Schematic diagram illustrating the tumor model and drug delivery schedule. Nude mice bearing MDA-MB-231 and HCC1937 xenografts transfected with sh-NC or sh-NAT10 were treated with olaparib (i.p.) following the indicated timeline. (B–D) Tumor volume (B), representative images (C), and the weight (D) of tumors of each group (*n* = 5) after the tumors were surgically dissected. (E) Body weight curves of mice in each group. (F) Western blotting for γ-H2AX expression level after treatment in tumor tissues. Data are presented as mean ± SD. ∗∗∗*p* < 0.001 and ∗∗∗∗*p* < 0.0001. NAT10, N-Acetyltransferase 10; TNBC, triple-negative breast cancer; DSBs, double-strand breaks.

In TNBC cells, NAT10 inhibition enhanced sensitivity to olaparib. To further investigate this effect *in vivo*, MDA-MB-231 and HCC1937 cells transfected with sh-NC or sh-NAT10 were implanted into the mammary fat pads of female nude mice. The mice were divided into four groups: sh-NC, sh-NC + olaparib, sh-NAT10, and sh-NAT10 + olaparib (Figure 3A). Tumor volumes were measured at designated time points. Notably, in response to olaparib treatment, mice bearing sh-NAT10 tumors exhibited significantly smaller tumor volumes compared to the sh-NC group (Figures 3B and 3C). Tumor weights in each group are shown in Figure 3D. Additionally, there were no significant differences in the average body weight among the groups, indicating that all treatment regimens were well-tolerated (Figure 3E). Western blotting analysis revealed that, following olaparib treatment, sh-NAT10 tumors exhibited higher levels of γ-H2AX protein expression compared to the sh-NC group (Figure 3F). The histological assessment of IHC staining revealed that sh-NAT10 tumors exhibited higher protein expression levels of γ-H2AX and cleaved caspase-3, as well as a lower number of Ki67-positive cells, compared to the sh-NC group following olaparib treatment (Figures S7A–S7C).

In summary, NAT10 knockout *in vivo* enhances the sensitivity of TNBC to olaparib and potentiates olaparib-induced DSBs.

### N-acetyltransferase 10 knockout sensitized triple-negative breast cancer cells to olaparib in an N4-acetylcytidine-dependent manner

To investigate whether the effect of NAT10 on olaparib sensitivity in TNBC depended on its ac4C activity, sh-NAT10 cells were used to express either wild-type NAT10 or the acetyltransferase-deficient mutant NAT10 (G641E).^45^^,^^55^ Western blot results confirmed efficient NAT10 knockdown and validated the re-expression of wild-type and mutant NAT10 (Figure S8A). Dot blot analysis further showed that NAT10 knockdown significantly reduced ac4C levels, which were rescued by wild-type NAT10 but not by the G641E mutant (Figure S8B).

**Figure 4:**
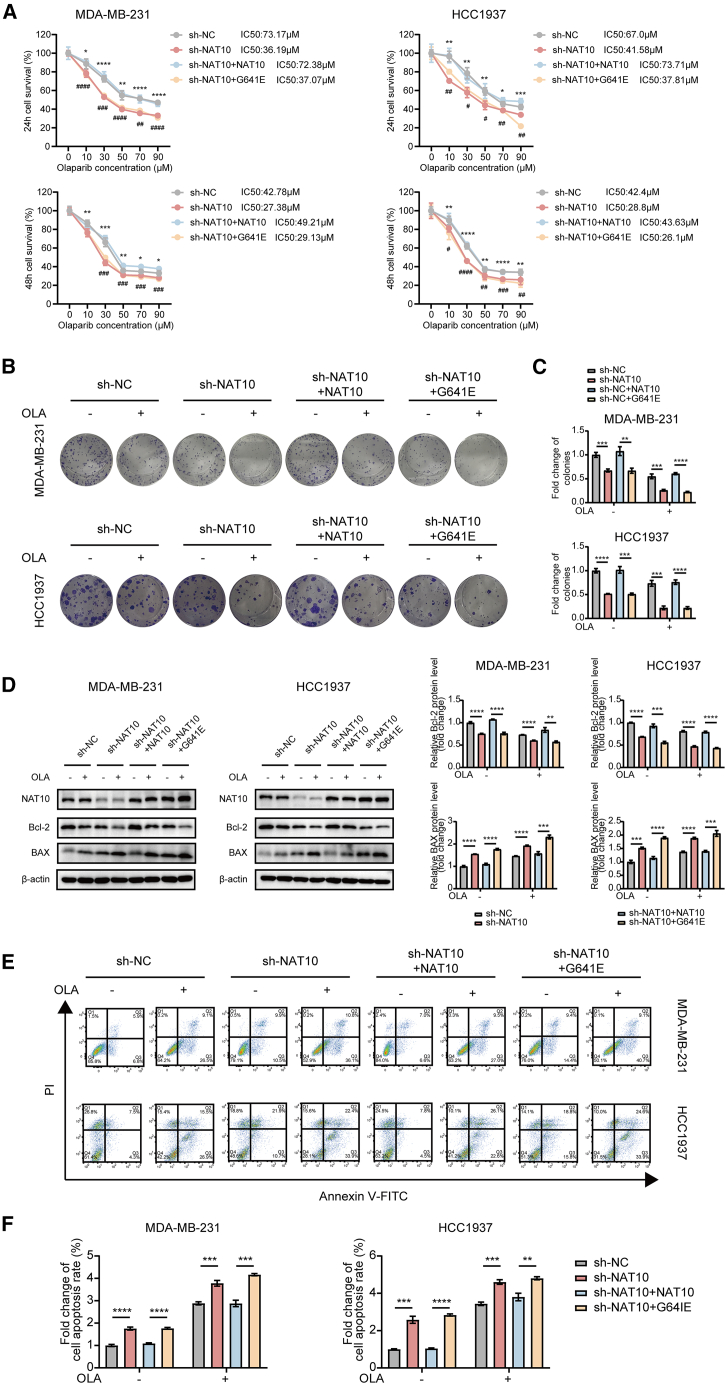
NAT10 knockout sensitized TNBC cells to olaparib in an ac4C-dependent manner (A) Cell survival assays were performed in MDA-MB-231 and HCC1937 cells to evaluate the sensitivity to olaparib under different conditions: sh-NC, sh-NAT10, sh-NAT10 + wild-type NAT10, and sh-NAT10 + G641E mutant NAT10. IC_50_ values were calculated at 24 and 48 h after treatment with increasing concentrations of olaparib (∗:sh-NC vs. sh-NAT10; #:sh-NAT10+NAT10 vs. sh-NAT10 + G641E). (B and C) Representative images (B) and quantification (C) of the colony formation assay in the above cells. (D) Western blot analysis to detect the expression levels of Bcl-2 and BAX proteins in the above cells. (E and F) Flow cytometry analysis (E) and quantification (F) of apoptosis in the above cells. Data are presented as mean ± SD. ∗*p* < 0.05, ∗∗*p* < 0.01, ∗∗∗*p* < 0.001 and ∗∗∗∗*p* < 0.0001. ^#^*p* < 0.05, ^##^*p* < 0.01, ^###^*p* < 0.001, and ^####^*p* < 0.0001. NAT10, N-Acetyltransferase 10; TNBC, triple-negative breast cancer; ac4C, N4- acetylcytidine.

As a result, the knockout of NAT10 enhanced the sensitivity of TNBC cells to olaparib, as indicated by decreased IC_50_ values in both MDA-MB-231 and HCC1937 cells. Re-expression of wild-type NAT10 reversed this sensitivity, while the mutant NAT10 failed to do so (Figure 4A). Colony formation assays demonstrated fewer colonies in NAT10-out cells treated with olaparib, and this phenotype was rescued by wild-type NAT10 but not by the G641E mutant (Figures 4B and 4C). Furthermore, Western blot analysis revealed that NAT10 knockdown increased pro-apoptotic BAX levels while decreasing anti-apoptotic Bcl-2 levels in response to olaparib treatment. Wild-type NAT10 restored these protein levels, whereas the G641E mutant did not (Figure 4D). Finally, flow cytometry analysis showed increased apoptosis in NAT10-knockdown cells upon olaparib treatment, which was rescued by wild-type but not mutant NAT10 (Figures 4E and 4F).

These findings demonstrate that NAT10 knockout sensitized TNBC cells to olaparib in an ac4C-dependent manner, underscoring the critical role of NAT10's RNA ac4C modification function in modulating drug sensitivity.

### N-acetyltransferase 10 suppression enhances olaparib-induced double-strand breaks in an N4-acetylcytidine-dependent manner

**Figure 5:**
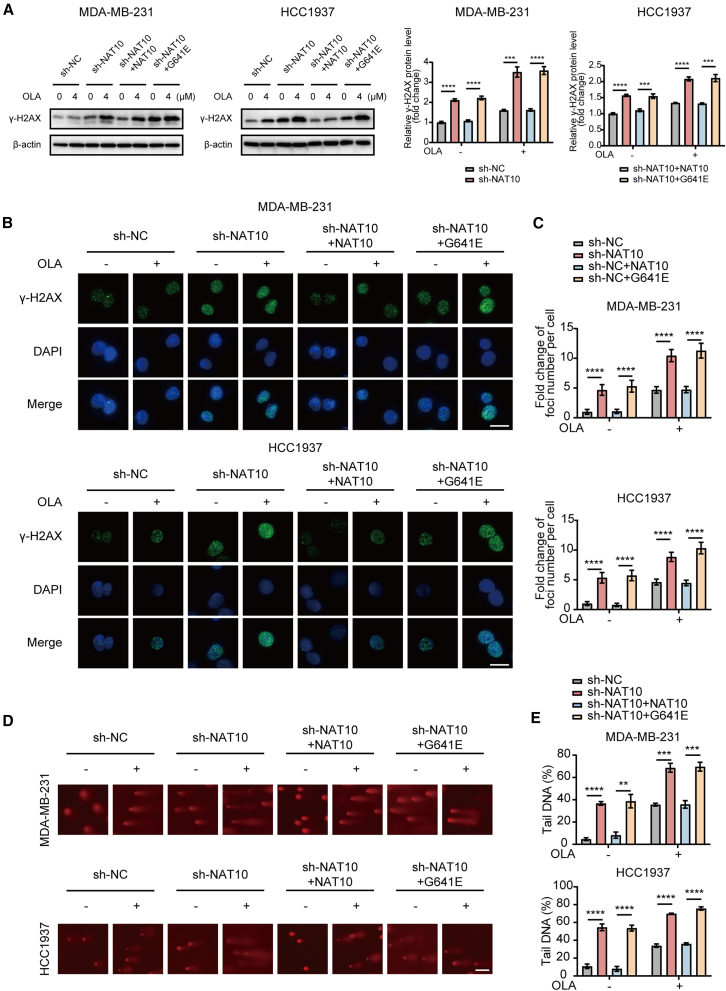
NAT10 suppression enhances olaparib-induced DSBs in an ac4C-dependent manner (A) Western blot analysis to evaluate the expression levels of γ-H2AX in MDA-MB-231 and HCC1937 cells under different treatment conditions. Cells were treated with or without olaparib at varying concentrations (0 and 4 μM), and the effects were compared among sh-NC, sh-NAT10, sh-NAT10+NAT10, and sh-NAT10 + G641E groups. (B and C) Representative immunofluorescence images (B) and quantification (C) of γ-H2AX foci formation in the above cells. γ-H2AX foci are stained green, and nuclei are counterstained with DAPI (blue). Merged images highlighting the colocalization of γ-H2AX foci within the nuclei. Scale bar: 20 μm. (D and E) Comet assay images (D) and quantification of tail DNA percentage (E) in the above cells. Scale bar: 50 μm. Data are presented as mean ± SD. ∗∗*p* < 0.01, ∗∗∗*p* < 0.001 and ∗∗∗∗*p* < 0.0001. NAT10, N-Acetyltransferase 10; DSBs, double-strand breaks; ac4C, N4-acetylcytidine.

Building upon the previous findings, we further explored whether the enhancement of olaparib-induced DSBs in NAT10-deficient TNBC cells depended on the ac4C modification activity of NAT10. In TNBC cells, the knockout of NAT10 significantly amplified DSBs induced by olaparib, as indicated by elevated levels of γ-H2AX and increased foci formation in MDA-MB-231 and HCC1937 cells. The re-expression of wild-type NAT10 effectively reversed these changes, whereas the G641E mutant was unable to achieve similar effects (Figures 5A–5C). Furthermore, comet assay results demonstrated that the knockout of NAT10 increased the percentage of comet tail DNA upon olaparib treatment, a phenotype that was rescued by wild-type NAT10 but remained unaffected by the G641E mutant (Figures 5D and 5E).

Overall, the enhancement of olaparib-induced DSBs by the loss of NAT10 in TNBC cells critically depends on the ac4C modification activity mediated by NAT10.

### RAD51 was the target of N-acetyltransferase 10 and was regulated by N4-acetylcytidine modification

**Figure 6:**
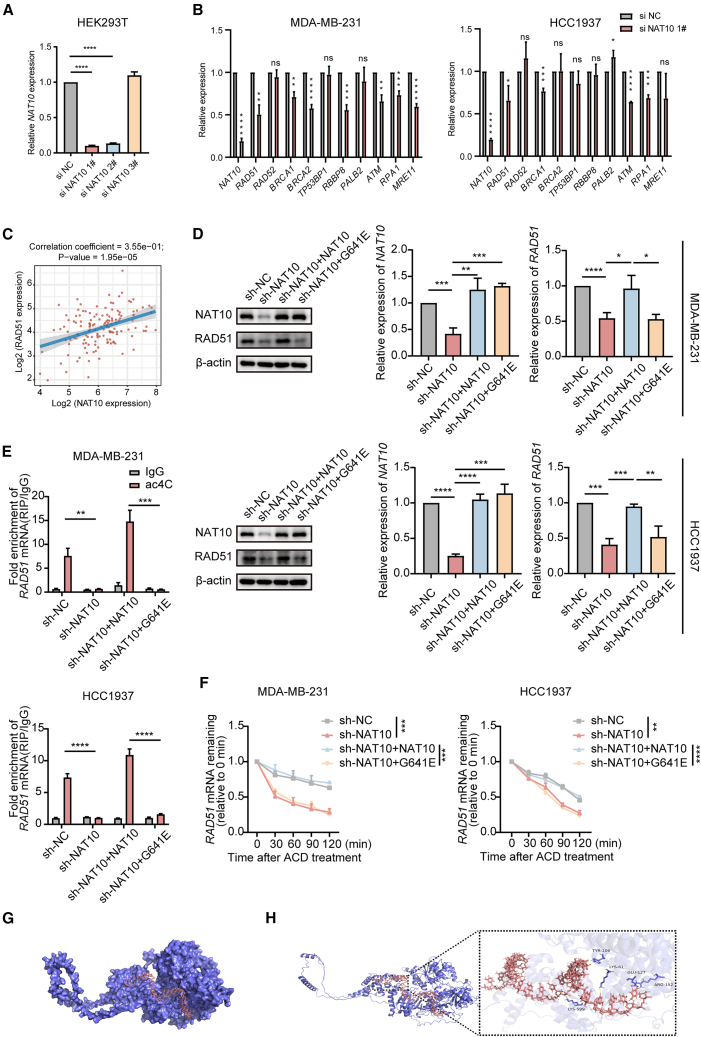
RAD51 was the target of NAT10 and was regulated by ac4C modification (A) Expression of *NAT10* in HEK293T cells transfected with siRNAs analyzed using qRT-PCR. (B) qRT-PCR examination of the expression of HR-associated key genes in MDA-MB-231 and HCC1937 cells transfected with a *NAT10*-specific siRNA or scrambled siRNA. (C) Correlation analysis between NAT10 and RAD51 expression levels among TNBC samples from the TCGA database. The scatterplot illustrates the correlation coefficients and *p*-values. (D) Western blot and qRT-PCR analysis of NAT10 and RAD51 levels in MDA-MB-231 and HCC1937 cells under different conditions, including sh-NC, sh-NAT10, sh-NAT10+NAT10, and sh-NAT10 + G641E. (E) acRIP-qRT-PCR of ac4C-modified *RAD51* mRNA in the above cells. (F) mRNA stability assay to evaluate *RAD51* mRNA degradation over time in the above cells treated with ACD. (G) The overall docking model with the NAT10 protein in purple and *RAD51* mRNA in red. (H) A detailed view of the interaction site, highlighting hydrogen bond interactions (yellow dotted lines). Data are presented as mean ± SD. ∗*p* < 0.05, ∗∗*p* < 0.01, ∗∗∗*p* < 0.001 and ∗∗∗∗*p* < 0.0001. NAT10, N-Acetyltransferase 10; ac4C, N4- acetylcytidine; HR, homologous recombination; TNBC, triple-negative breast cancer; TCGA, The Cancer Genome Atlas; ACD, actinomycin D.

Given the significant findings regarding the effects of NAT10 on olaparib sensitivity and DNA damage in TNBC cells, we aimed to explore the specific underlying molecular mechanisms, particularly focusing on potential targets associated with NAT10-mediated ac4C modification. Consequently, we identified the candidates that were regulated by this modification and were involved in both HR and the sensitivity of TNBC to olaparib. We measured *NAT10* mRNA levels in HEK293T cells transfected with three different siRNAs. Since *NAT10* siRNA 1# exhibited the most significant downregulation, we proceeded with it for subsequent experiments (Figure 6A). Through an extensive literature search, we meticulously identified ten key genes that were critically involved in HR repair pathways. We analyzed various studies highlighting the roles of these genes in DNA repair mechanisms. We focused on genes with significant relevance to breast cancer biology and HR repair efficiency. Following this selection, we investigated the regulatory relationships between *NAT10* and the identified genes. Based on the results of the qRT-PCR, after *NAT10* knockdown, *RAD51* showed a significant decrease in expression (Figure 6B). In a previous study conducted by our research group, RNA sequencing was performed on BT-549 cells transfected with siNC or siNAT10. The analysis revealed that *NAT10* knockdown resulted in a reduction in *RAD51* expression levels (*p* < 0.0001).^56^ In addition, the results of The Cancer Genome Atlas (TCGA)-BRCA: https://portal.gdc.cancer.gov/ showed that NAT10 was positively correlated with RAD51 in TNBC (Figure 6C). To further validate the role of ac4C modification in the regulation of RAD51 by NAT10, we assessed RAD51 expression levels under conditions of NAT10 knockout and re-expression of either wild-type NAT10 or the G641E mutant NAT10. The results indicated that in both MDA-MB-231 and HCC1937 cells, RAD51 protein levels were significantly decreased in NAT10-depleted cells, while this effect was reversed by the re-expression of wild-type NAT10 but not its mutants (Figure 6D). Similarly, qRT-PCR analysis revealed that in sh-NAT10 cells, the re-expression of wild-type NAT10 successfully restored *RAD51* mRNA levels to baseline, while the G641E mutant had no such effect (Figure 6D). These findings indicate that NAT10 regulates the expression of RAD51 by ac4C modification. As expected, treatment with remodelin led to a dose-dependent and significant reduction in RAD51 expression levels (Figure S9A).

Furthermore, we performed an ac4C-RIP assay using an ac4C antibody followed by qRT-PCR for the predicted regions of ac4C (PACES (rnanut.net)). Using specific primers designed for the predicted ac4C-harboring regions of RAD51, qRT-PCR data showed that NAT10 knockout downregulated the ac4C modification of *RAD51* mRNA, whereas the overexpression of wild-type NAT10, but not the G641E mutant, could rescue this modification (Figure 6E).

We further examined whether NAT10 affected the stability of *RAD51* mRNA. Indeed, the loss of NAT10 markedly reduced the stability of *RAD51* transcripts, whereas the overexpression of wild-type NAT10, but not the mutant NAT10, mitigated this effect (Figure 6F). Moreover, overexpression of NAT10 was observed to enhance the stability of *RAD51* mRNA (Figure S9B).

To verify the relationship, we performed protein-RNA docking between NAT10 and *RAD51* mRNA (Figures 6G and 6H). The results showed that NAT10 docked with *RAD51* mRNA with an affinity score of −468.8 kcal/mol. The high-affinity docking results indicated that this interaction is stable and possible at the molecular level.

These findings underscore that RAD51 regulation relies on NAT10-driven ac4C modifications.

### N-acetyltransferase 10 reduces olaparib sensitivity in triple-negative breast cancer cells by regulating the N4-acetylcytidine modification of RAD51

**Figure 7:**
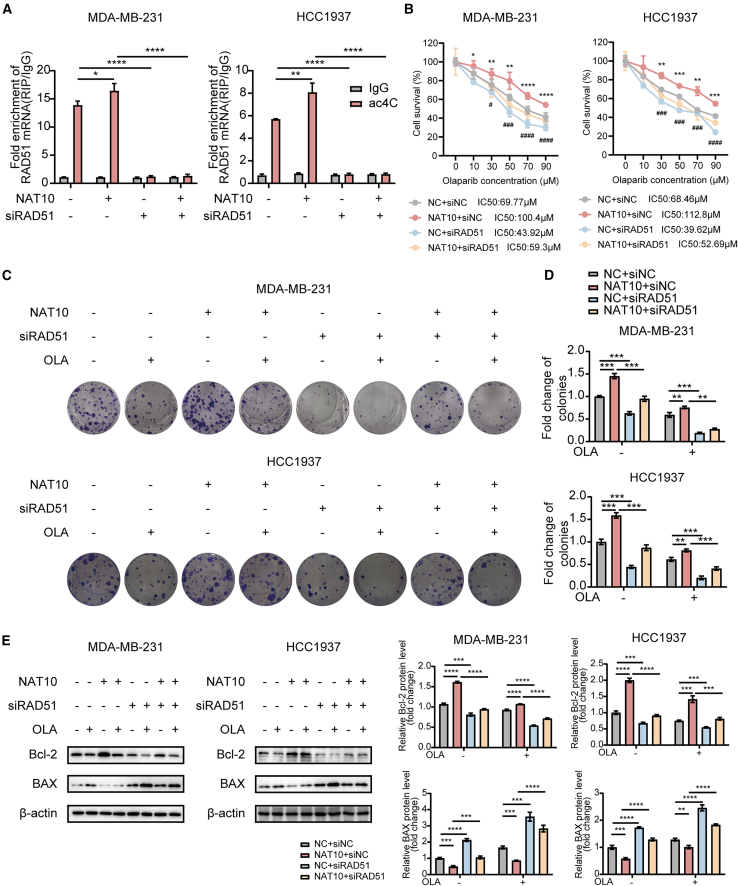
NAT10 reduces olaparib sensitivity in TNBC cells by regulating the ac4C modification of RAD51 (A) acRIP-qRT-PCR analysis to assess the enrichment of ac4C-modified *RAD51* mRNA in MDA-MB-231 and HCC1937 cells. Comparisons were made among cells with NAT10 overexpression and/or siRAD51 knockdown. (B) Cell viability assay of MDA-MB-231 and HCC1937 cells treated with different concentrations of olaparib in the above cells (∗:NC + siNC vs. NAT10+siNC; #:NAT10+siNC vs. NAT10+siRAD51). (C and D) Representative images (C) and quantification (D) of colony formation assay in MDA-MB-231 and HCC1937 cells treated with different concentrations of olaparib in the above cells. (E) Western blot analysis to detect the expression levels of Bcl-2 and BAX proteins in MDA-MB-231 and HCC1937 cells treated with different concentrations of olaparib in the above cells. Data are presented as mean ± SD. ∗*p* < 0.05, ∗∗*p* < 0.01, ∗∗∗*p* < 0.001 and ∗∗∗∗*p* < 0.0001. ^#^*p* < 0.05, ^###^*p* < 0.001 and ^####^*p* < 0.0001. NAT10, N-Acetyltransferase 10; TNBC, triple-negative breast cancer; ac4C, N4- acetylcytidine.

We next investigated whether the effects of NAT10 on olaparib sensitivity in TNBC cells were RAD51 ac4C modification dependent. Overexpression of NAT10 increased the ac4C modification of *RAD51* mRNA, as demonstrated by ac4C-RIP analysis, while siRNA-mediated knockdown of RAD51 diminished this effect (Figure 7A). Functionally, the overexpression of NAT10 inhibited olaparib sensitivity in both MDA-MB-231 and HCC1937 cells, as evidenced by a marked elevation in the IC_50_ values of olaparib. However, simultaneous knockdown of RAD51 partially restored TNBC sensitivity to olaparib (Figure 7B). Further supporting these findings, colony formation assays showed that NAT10 overexpression significantly enhanced the number of colonies in olaparib-treated TNBC cells, while RAD51 knockdown in NAT10-overexpressing cells partially restored colony-forming ability (Figures 7C and 7D).

Similarly, it was revealed that NAT10 overexpression decreased BAX levels and increased Bcl-2 levels in TNBC cells treated with olaparib, indicating diminished apoptosis. Co-knockdown of RAD51 reversed these protein expression changes (Figure 7E).

In summary, our results confirm that NAT10 reduces olaparib sensitivity in TNBC cells by regulating the ac4C modification of RAD51.

### N-acetyltransferase 10 inhibits olaparib-induced double-strand breaks by regulating the N4-acetylcytidine modification of RAD51

**Figure 8:**
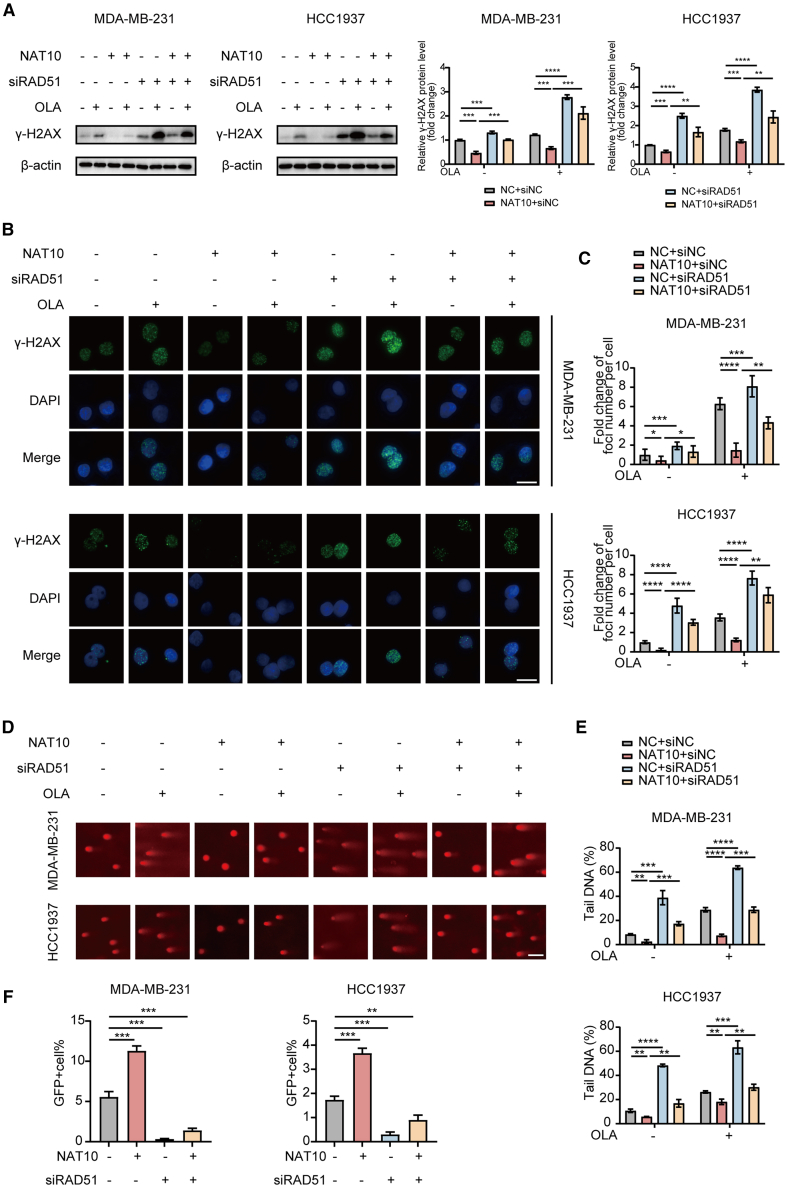
NAT10 inhibits olaparib-induced DSBs by regulating the ac4C modification of RAD51 (A) Western blot analysis to evaluate the expression levels of γ-H2AX in MDA-MB-231 and HCC1937 cells with or without olaparib treatment. Comparisons were made among cells with NAT10 overexpression and/or siRAD51 knockdown. (B and C) Representative images (B) and quantification (C) of γ-H2AX foci formation in the above cells. Scale bar: 20 μm. (D and E) Comet assay images (D) and quantification of tail DNA percentage (E) in the above cells. Scale bar: 50 μm. (F) The frequency of GFP-positive (GFP+) cells of HR-mediated DSBs repair in the above cells. Data are presented as mean ± SD. ∗*p* < 0.05, ∗∗*p* < 0.01, ∗∗∗*p* < 0.001 and ∗∗∗∗*p* < 0.0001. NAT10, N-Acetyltransferase 10; DSBs, double-strand breaks; ac4C, N4- acetylcytidine; HR, homologous recombination.

In order to investigate whether NAT10 inhibits olaparib-induced DSBs through the ac4C modification of RAD51, we measured the levels of γ-H2AX. It was revealed that NAT10 overexpression decreased γ-H2AX levels in olaparib-treated MDA-MB-231 and HCC1937 cells. However, co-silencing RAD51 partially reversed this reduction, restoring γ-H2AX levels (Figure 8A). Additionally, immunofluorescence staining showed the same observations (Figures 8B and 8C).

Consistent with these results, comet assays indicated that NAT10 overexpression reduced the percentage of tail DNA, indicative of decreased DSBs, in olaparib-treated cells. Co-silencing RAD51 reversed this phenotype, increasing the percentage of tail DNA in both MDA-MB-231 and HCC1937 cells (Figures 8D and 8E).

Further, HR reporter assays showed that NAT10 overexpression enhanced HR repair efficiency, as evidenced by an increased percentage of GFP-positive cells, while RAD51 knockdown abolished this enhancement (Figure 8F).

These results collectively demonstrate that NAT10 inhibits olaparib-induced DSBs by promoting RAD51-mediated HR repair through ac4C modification.

### Remodelin enhanced the sensitivity to olaparib and enhanced olaparib-induced double-strand breaks in triple-negative breast cancer cells

**Figure 9:**
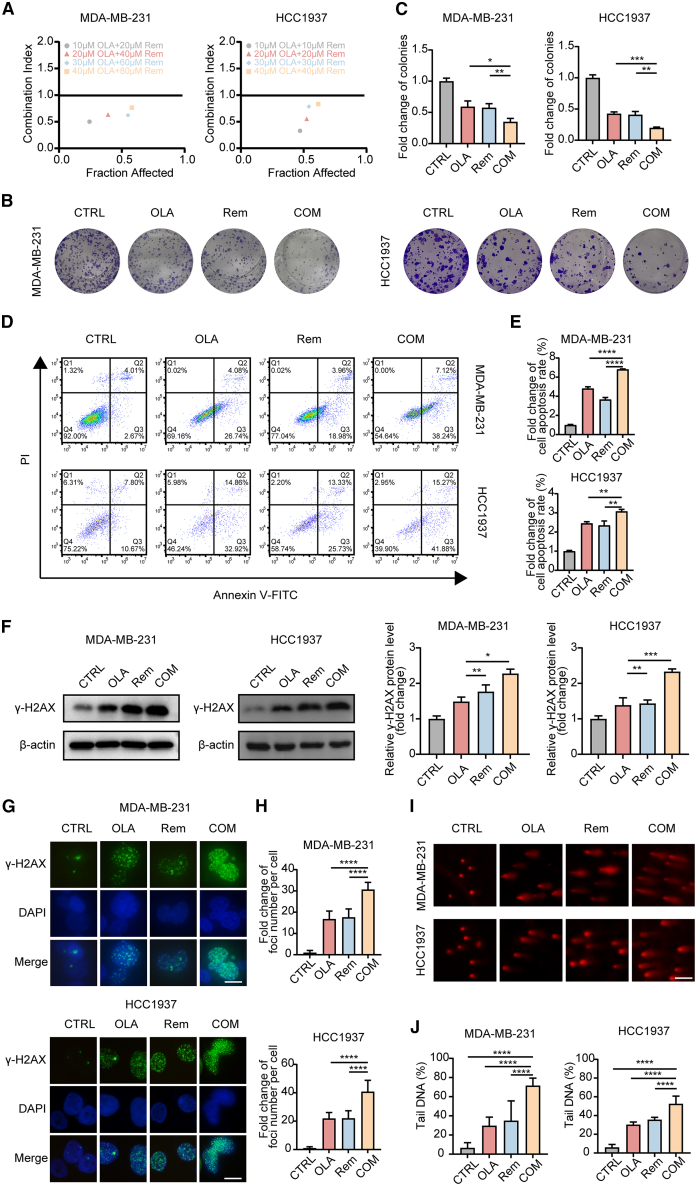
Remodelin enhanced the sensitivity to olaparib and enhanced olaparib-induced DSBs in TNBC cells (A) Scatterplots representing the CI for MDA-MB-231 and HCC1937 cell lines treated with the combination of olaparib and remodelin. The CI values are plotted against the fraction affected, indicating the level of drug synergy, where a CI value less than 1 suggests synergism. (B and C) Representative images (B) and quantification (C) of colony formation assay in MDA-MB-231 and HCC1937 cells treated with DMSO (CTRL), olaparib (2 μM), remodelin (35 μM), or the combination of olaparib (2 μM) and remodelin (35 μM). (D and E) Flow cytometry analysis (D) and quantification (E) of apoptosis in MDA-MB-231 and HCC1937 cells treated with DMSO (CTRL), olaparib (2 μM), remodelin (35 μM), or the combination of olaparib (2 μM) and remodelin (35 μM). (F) Western blot analysis was performed to determine the levels of γ-H2AX in MDA-MB-231 and HCC1937 cell lines treated with DMSO (CTRL), olaparib (2 μM), remodelin (35 μM), or a combination of olaparib (2 μM) and remodelin (35 μM). (G and H) Representative images (G) and quantification (H) of γ-H2AX foci formation in the above cells. Scale bar: 20 μm. (I and J) Comet assay images (I) and quantification of tail DNA percentage (J) in the above cells. Scale bar: 50 μm. Data are presented as mean ± SD. ∗*p* < 0.05, ∗∗*p* < 0.01, ∗∗∗*p* < 0.001 and ∗∗∗∗*p* < 0.0001. DSBs, double-strand breaks; TNBC, triple-negative breast cancer; CI, combination index.

Since remodelin is a well-known inhibitor of NAT10, we investigated whether it can sensitize TNBC cells to olaparib. We measured the effects of the combination of remodelin and olaparib on cell growth in TNBC cell lines. The combination therapy significantly enhanced the inhibitory effects of olaparib. Importantly, this effect was not merely additive but synergistic, evidenced by combination index values of less than 1 (Figures 9A and S10A), which suggested a more potent inhibitory effect compared to either drug alone. To confirm these findings, we conducted a colony-formation assay. Interestingly, the results showed that combination therapy with remodelin (35 μM) and olaparib (2 μM) markedly reduced the number of colonies, surpassing the effects of either remodelin or olaparib alone (Figures 9B, 9C, S10B, and S10C).

In addition to growth inhibition, we explored the effects of the combination therapy on cell apoptosis. The Annexin V-FITC/PI dual staining method showed that, compared to single drugs, the combination of remodelin (35 μM) and olaparib (2 μM) induced markedly higher levels of cell apoptosis in TNBC cell lines (Figures 9D, 9E, S10D, and S10E).

To delve deeper into the mechanisms underlying this enhanced efficacy, we investigated the effects of the combination therapy on DNA damage. Specifically, Western blotting was conducted to assess the levels of the DNA damage marker γ-H2AX. The results indicated that in TNBC cells, the combination of remodelin (35 μM) and olaparib (2 μM) significantly increased the abundance of γ-H2AX compared to the single-dose group and control group (Figures 9F and S10F). Subsequently, we used immunofluorescence staining, which confirmed the results of Western blotting. Remarkably, the combination of remodelin and olaparib notably increased γ-H2AX nuclear foci formation compared to single agent treatment (Figures 9G, 9H, S10G, and S10H). The comet assay showed similar results, with a higher percentage of DNA tails in the combination therapy group compared to single drug groups (Figures 9I, 9J, S10I, and S10J).

Altogether, our data demonstrate that the combination of remodelin and olaparib exerts a synergistic effect in TNBC cells by enhancing growth inhibition, promoting apoptosis, and inducing DNA damage, surpassing the efficacy of either drug alone.

### Co-administration of remodelin and olaparib exerted synergistic effects *in vivo*

**Figure 10:**
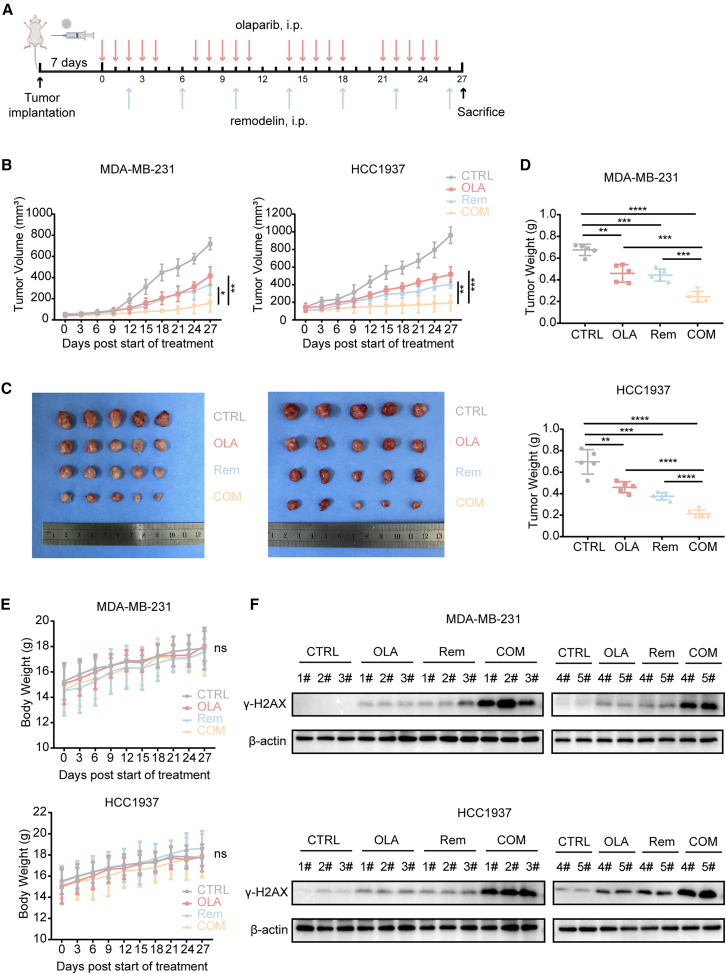
Co-administration of remodelin and olaparib showed a synergistic effect *in vivo* (A) Schematic diagram illustrating the tumor model and drug delivery schedule. Nude mice bearing MDA-MB-231 and HCC1937 xenografts were treated with olaparib (i.p.) and remodelin (i.p.) following the indicated timeline. Mice were sacrificed at the end of the treatment period for analysis. (B) Tumor volume of nude mice bearing MDA-MB-231 and HCC1937 xenografts. Tumor-bearing mice were randomized into 4 groups (*n* = 5) treated with either vehicle, a single agent, or combination therapy. (C) Tumor tissue in each group of mice. (D) Tumor weight of mice in each group. (E) Body weight curves of mice treated with either vehicle, a single agent, or combination therapy. (F) Western blotting for γ-H2AX expression level after treatment with either vehicle, a single agent, or combination therapy in tumor tissues from MDA-MB-231 and HCC1937 breast cancer xenografts. Data are presented as mean ± SD. ∗*p* < 0.05, ∗∗*p* < 0.01, ∗∗∗*p* < 0.001 and ∗∗∗∗*p* < 0.0001.

Since NAT10 inhibition enhanced the sensitivity to olaparib in cell culture, we compared the effects of the combination of remodelin and olaparib with that of remodelin and olaparib alone on the growth of tumor xenografts in nude mice. We investigated the xenografts of the BRCA-profit TNBC cell line model MDA-MB-231 and the BRCA-defect TNBC cell line model HCC1937. To reflect the original microenvironment, MDA-MB-231 and HCC1937 cells were directly implanted into the mammary fat pad of female nude mice. After one week of inoculation, mice were treated with remodelin, olaparib, or their combination, and the tumor volume was measured at designated time intervals (Figure 10A). Compared to the carrier control, treatment with remodelin or olaparib significantly inhibited tumor growth. The combination of remodelin and olaparib had a greater inhibitory effect on tumor growth compared to remodelin and olaparib alone (Figures 10B and 10C). The tumor weight of each group was shown in Figure 10D.

There was no significant difference in average body weight between combination therapy and single therapy, indicating that all treatment regimens were well-tolerated (Figure 10E). The endpoint study determined that the protein levels of DNA damage marker γ-H2AX were elevated in the combination group (Figure 10F). Tumor xenografts were collected for histological assessment with IHC staining. Remodelin upregulated γ-H2AX and cleaved caspase 3, which were also upregulated after treatment with the combination remodelin and olaparib (Figures S11A and S11B). Compared with monotherapy, combination therapy decreased the number of Ki67-positive cells (Figure S11C).

In conclusion, NAT10 may regulate DNA damage repair in a cell- or tissue-specific manner. These observations may provide a deeper insight into the subset of TNBC that may be effectively treated with the combination of remodelin and olaparib.

## Discussion

With the development of various inhibitors related to DNA repair pathways, synthetic lethal therapies have gradually become an emerging research field in the treatment of cancer.^57^^,^^58^ The PARP inhibitor olaparib is the first drug related to the concept of “synthetic lethal.”^11^^,^^59^ Although monotherapy with PARP inhibitors has shown promising therapeutic effects, resistance and toxic side effects still need further studies.^10^^,^^15^^,^^60^ In the present study, we aim to evaluate the impact of NAT10 on the sensitivity to olaparib in TNBC and its underlying mechanism.

The negative regulatory factor Circ-PPID of trastuzumab resistance is significantly downregulated in trastuzumab-resistant cells and tissues. Circ-PPID directly binds to NAT10 in the nucleus, blocking the interaction between NAT10 and *HER2* mRNA, reducing ac4C modification on HER2 exon 25, and leading to *HER2* mRNA attenuation. These data provide promising therapeutic directions for overcoming trastuzumab resistance in clinical settings.^61^ In addition, NAT10 was shown to promote the maintenance of stemness among colon cancer cells and enhance chemical resistance by stabilizing *NANOGP8* mRNA. The inhibitory effect of remodelin on NAT10 can improve the efficacy of chemotherapy and hinder colon cancer progression.^62^ Wu et al. found that the inhibition of NAT10 reduced doxorubicin resistance in breast cancer by reversing epithelial-mesenchymal transition (EMT).^63^ Similarly, NAT10 induced doxorubicin resistance in hepatocellular carcinoma by promoting EMT.^64^ NAT10 stabilizes the mRNA expression of the key DDR gene *AHNAK*, protecting it from exonuclease degradation and further enhancing the efficiency of the NHEJ repair pathway. Additionally, cisplatin can upregulate NAT10 transcription by activating the NF-κB signaling pathway, further amplifying its role in DDR.^45^ These phenomena indicate that remodelin may improve the efficacy of chemotherapy for breast cancer and hepatocellular carcinoma. Moreover, HCC cell lines Huh-7 and Hep3B cells treated with NAT10-siRNA and remodelin were sensitive to lenvatinib within 24 and 48 h.^48^ However, the mechanisms underlying these observations deserve further studies. Our study indicated that NAT10 inhibition enhances the sensitivity of TNBC cells to olaparib in an ac4C-dependent manner, unveiling a potential mechanism behind olaparib resistance in TNBC.

The role of NAT10 in DNA damage repair has been well characterized. Hydrogen peroxide (H2O2) or cisplatin significantly increases *NAT10* mRNA levels in a dose- and time-dependent manner. Both H2O2 and cisplatin stimulate the transcriptional activity of NAT10 via upstream sequences of its promoter, which interact with specific nuclear proteins. Additionally, exogenous expression of NAT10 enhances cell survival in the presence of H2O2 or cisplatin. These findings suggest that NAT10 may be involved in the DNA damage response and increased cellular resistance to genotoxic stress.^53^ Zhang et al. demonstrated that MORC2 enhances its ATPase and chromatin remodeling activities through interaction with PARP1 and subsequent PARylation. MORC2 stabilizes PARP1 via NAT10-mediated acetylation, thereby playing a critical role in the DNA damage response, promoting DNA repair, and increasing cellular resistance to genotoxic stress.^52^ Additionally, upon DNA damage due to chemotherapy or radiotherapy, NAT10 translocates from the nucleolus to the nucleoplasm, significantly increasing the acetylation of MORC2 at lysine 767 (K767Ac). This modification disrupts MORC2’s interaction with phosphorylated histone H3 at threonine 11 (H3T11), inhibiting CDK1 and cyclin B1 transcription. The activation of the G2 checkpoint slows or halts the cell cycle in late DNA synthesis, allowing time for DNA repair and preventing mitosis with unrepaired chromatin. These findings uncover the role of MORC2 in DNA damage-induced G2 checkpoint via NAT10-mediated acetylation and suggest a potential therapeutic strategy to enhance the sensitivity of breast cancer cells to DNA-damaging treatments by targeting NAT10.^49^ The team also unveiled that NAT10 undergoes covalent PARylation following DNA damage, with poly(ADP-ribose) polymerase 1 (PARP1) catalyzing PARylation at three conserved lysine residues (K1016, K1017, and K1020). Notably, the mutation of these residues, inhibition of PARP1, or depletion of PARP1 impairs the nucleoplasmic translocation of NAT10 after DNA damage. PARP1 knockdown or inhibition, along with the expression of a PARylation-deficient NAT10 (K3A) mutant, inhibits the co-localization of NAT10 and MORC2, resulting in decreased MORC2 acetylation at lysine 767 and increased cellular sensitivity to DNA-damaging agents. These findings reveal that the PARP1-mediated PARylation of NAT10 is crucial for its nucleoplasmic translocation and function in the DNA damage response, providing insights into posttranslational modification-driven cellular response to DNA damage.^51^ NAT10-mediated ac4C in mRNA can increase mRNA stability and translation, but NAT10 knockdown enhances the repair of UVB-induced DNA damage by promoting the mRNA stability of *DDB2*. This finding illuminates the role of NAT10 in DNA damage repair.^54^ Our data demonstrated that compared to control cells, TNBC cells with NAT10 knockout showed an increased susceptibility to DSBs following treatment with olaparib. Furthermore, we identified that among various DNA damage response genes, the *RAD51* gene was regulated in a NAT10-ac4C-dependent manner. Both NAT10 knockout and NAT10 inhibitor reduced RAD51 expression in TNBC cells. Mechanistically, NAT10 inhibits olaparib-induced DSBs by regulating the ac4C modification of RAD51, suggesting that combination therapy with NAT10 inhibitors and PARP inhibitors is an innovative treatment strategy for TNBC.

Recent advances in cancer treatment have highlighted the potential of combination therapies to enhance therapeutic efficacy. The combination of PARP inhibitors with immune checkpoint inhibitors, such as anti-PD-1 antibodies, demonstrated significant potential in treating patients with *BRCA1/2*-mutant cancers. This effect arises from an increased mutational burden, activation of the cGAS-STING signaling pathway, and enhanced CD8^+^ T cell infiltration and activation.^65^^,^^66^^,^^67^^,^^68^ In the TOPACIO trial, the combination therapy of niraparib and pembrolizumab achieved an overall response rate (ORR) of 18% in the ovarian cancer cohort. Notably, patients without tumor BRCA mutations or non-HRD cancers exhibited a higher response rate than what would be expected from either drug used as monotherapy.^69^ Furthermore, the MEDIOLA trial revealed that the combination of olaparib and durvalumab resulted in a 50% disease control rate at 28 weeks among patients with *gBRCA1/2*-mutant metastatic breast cancer.^70^ Nevertheless, the effectiveness of this combination in patients with acquired resistance to PARP inhibitors needs further studies. Furthermore, the combination of PARP inhibitors with XPO1 inhibitors or Bcl-2 inhibitors is being explored in TNBC clinical trials (NCT02419495 and NCT05358639). In light of the demonstrated synergistic effects of topoisomerase I (TOP1) inhibitors and PARP inhibitors, coupled with their potential to achieve synthetic lethality, future studies are encouraged to investigate the combination of PARP inhibitors with antibody-drug conjugates (ADCs) that can effectively deliver TOP1 inhibitors, such as sacituzumab-govitecan or trastuzumab deruxtecan (T-DXd).^28^^,^^71^^,^^72^ Moreover, inhibitors targeting different stages of the cell cycle, such as ATM inhibitors, ATR inhibitors, WEE1 inhibitors, and CHK1 inhibitors, have been developed in recent years, and their combination with PARP inhibitors has demonstrated enhanced anti-cancer efficacy.^29^^,^^30^^,^^73^^,^^74^^,^^75^^,^^76^^,^^77^ By interfering with gene expression, nuclear localization, or the recruitment of HR proteins, inhibitors targeting pathways such as EGFR, IGF1R, VEGF, or the PI3K–AKT pathway can indirectly inhibit HR.^24^^,^^25^^,^^26^^,^^27^^,^^78^ The combination of these inhibitors with PARP inhibitors has demonstrated enhanced anti-tumor activity. Additionally, the indirect inhibition of HR can be achieved by targeting epigenetic regulators. For example, BET and BRD4 inhibitors suppress the transcription of key DDR genes, thereby enhancing the efficacy of PARP inhibitors.^79^^,^^80^^,^^81^ Inhibition of histone deacetylases and CDK1 and CDK12 cyclin-dependent kinases that regulate HR repair can also suppress HR and enhance the sensitivity to PARP inhibitors.^82^^,^^83^^,^^84^ Furthermore, HSP90 inhibitors destabilize HR proteins, such as RAD51, BRCA1, and BRCA2, induce HR deficiency, and promote sensitivity to PARP inhibitors.^85^^,^^86^ Given these encouraging developments in combination therapies, we investigated the synergistic potential of combining the NAT10 inhibitor remodelin with olaparib. In our study, remodelin was found to synergistically interact with olaparib based on the combination index. This combination was proven to be effective in suppressing cell proliferation, inducing apoptosis, and promoting the formation of DSBs *in vitro* and *in vivo*.

Our study highlighted the crucial role of NAT10 in maintaining homologous recombination through ac4C-mediated regulation of RAD51. NAT10 inhibition markedly depleted RAD51, which led to homologous recombination deficiency and enhanced the sensitivity of TNBC cells to olaparib. These results not only clarify the molecular interplay between NAT10 and RAD51 but also suggest that the concurrent use of PARP inhibitors and NAT10 inhibitors, such as remodelin, can vigorously treat TNBC. This combination therapy can enhance the efficacy of existing treatments and offer potential benefits for patients with TNBC, emphasizing the need for further clinical studies.

### Limitations of the study

This study has some limitations. First, PARP inhibitors have been approved for the treatment of various cancers, including ovarian,^87^^,^^88^^,^^89^^,^^90^^,^^91^ prostate,^92^^,^^93^ pancreatic,^94^ and breast cancers.^20^^,^^21^ However, our study is meticulously focused solely on breast cancer. Then, although extensive preclinical and clinical research has been conducted on a variety of PARP inhibitors within the realm of cancer research,^16^^,^^95^^,^^96^ our investigation centers exclusively on olaparib. Therefore, broader and more in-depth experimental studies will be necessary in the future to expand upon these findings.

## Resource availability

### Lead contact

Further information and requests for resources and reagents should be directed to and will be fulfilled by the lead contact, Da Pang (pangda@ems.hrbmu.edu.cn).

### Materials availability

This study did not generate new unique reagents.

### Data and code availability

• Data reported in this article will be shared by the lead contact upon request.
• This article does not report original code.
• Any additional information required to reanalyze the data reported in this article is available from the lead contact upon request.

## Acknowledgments

This work was supported by the 10.13039/501100001809National Natural Science Foundation of China [Grant Numbers: 82173235, 82202996, 82103325, 82073410, and 82272623]; Key R&D Plan Projects of Heilongjiang Province [Grant Numbers: JD22C004 and 2023ZX06C10]; the Project Nn10 of the Harbin Medical University Cancer Hospital [Grant Numbers: 102017-02]; and Spring Goose Support Program of Heilongjiang Province [Grant Numbers: CYQN24010].

## Author contributions

Conceptualization: D.P. and X.Z.; data curation: H.L., S.L., and G.L.; formal analysis: H.L. and X.M.; methodology: Y.G., Y.C., and X.C.; resources: X.C.; supervision: H.W. and Q.W.; validation: S.J.; writing-original draft: H.L.; writing-review and editing: H.L. and H.W.

## Declaration of interests

The authors declare no competing interests.

## STAR★Methods

### Key resources table

**Table:** 

REAGENT or RESOURCE	SOURCE	IDENTIFIER
**Antibodies**		

Anti-NAT10 antibody	Abcam	Cat#ab194297; RRID: AB_3696046
Anti-β-actin antibody	ZSGB-BIO	Cat#TA-09; RRID: AB_2636897
Anti-BAX antibody	Cell Signaling Technology	Cat#2772; RRID: AB_10695870
Anti-Bcl-2 antibody	Wanleibio	Cat#WL01556; RRID: AB_2904235
Anti-Phospho-Histone H2A.X (Ser139) (20E3) antibody	Cell Signaling Technology	Cat#9718; RRID: AB_2118009
Anti-RAD51 antibody	Abcam	Cat#ab133534; RRID: AB_2722613
HRP-conjugated Goat Anti-Mouse IgG(H + L)	Proteintech	Cat#SA00001-1; RRID: AB_2722565
HRP-conjugated Goat Anti-Rabbit IgG(H + L)	Proteintech	Cat#SA00001-2; RRID: AB_2722564
Anti-ac4C antibody	Abcam	Cat#ab252215; RRID: AB_2827750
Anti-cleaved caspase-3 antibody	Cell Signaling Technology	Cat#9664; RRID: AB_2070042
Anti-Ki-67 antibody	Cell Signaling Technology	Cat#9449; RRID: AB_2797703
HRP-conjugated anti-rabbit second antibodies	ZSGB-BIO	Cat#PV-6001; RRID: AB_2864333

**Critical commercial assays**		

Annexin V-FITC Apoptosis Detection Kit	Beyotime	C1062
Universal IF Toolkit	Abbkine	KTD107
Comet Assay Kit	KeyGen Bio-Tech	KGA1302
EpiTM ac4C immunoprecipitation kit	Epibiotek	R1815

**Experimental models: Cell lines**		

MDA-MB-231	ATCC	HTB-26
BT549	ATCC	HTB-122
HCC1937	ATCC	CRL-2336
MDA-MB-436	ATCC	HTB-130
HEK293T	ATCC	CRL-3216

**Experimental models: Organisms/strains**		

Mouse:BALB/cnude	Liaoning Changsheng biotechnology co., Ltd.	N/A

**Oligonucleotides**		

shRNA targeting sequence:NAT10: GGAAUAUGGUGGACUAUCATT	This paper	N/A
shRNA targeting sequence:NC: UUCUCCGAACGUGUCACGUTT	This paper	N/A
siRNA targeting sequence:NAT10 1#: GGAAUAUGGUGGACUAUCATT	This paper	N/A
siRNA targeting sequence:NAT10 2#: GUACUCCAAUAUCUUUGUUTT	This paper	N/A
siRNA targeting sequence:NAT10 3#: GAGUACUGUUGCACUCACATT	This paper	N/A
siRNA targeting sequence:RAD51: GCAGUGAUGUCCUGGAUAATT	This paper	N/A
Primers for *NAT10*, *RAD51*, *RAD52*, *BRCA1*, *BRCA2*, *TP53BP1*, *RBBP8*, *PALB2*, *ATM*, *RPA1*, *MRE11*, *ACTB* and *RAD51* (ac4C)	This paper	See Table S1

**Software and algorithms**		

GraphPad 10.1	GraphPad Software	www.graphpad.com
casp1.2.3b2	CASP Software	www.bio-launching.com
HEX8.0	Hex Protein Docking Software	https://hex.loria.fr/
Pymol	DeLano Scientific LLC	https://pymol.org

### Experimental model and study participant details

#### Animals

Six-to-eight-week-old female nude mice were purchased from Beijing Weitong Lihua Experimental Animal Technology Co., Ltd. All animals were housed in a specific-pathogen-free facility at the Medical Experimental Animal Center of the Second Affiliated Hospital of Harbin Medical University, with free access to food and water under constant temperature conditions. All animal experiments were approved by the Medical Experimental Animal Care Committee of the Second Affiliated Hospital of Harbin Medical University (Approval no. SYDW2019-127 and SYDW2025-025) and were performed in accordance with the guidelines set forth by the National Institutes of Health for animal care and ethical standards. All mice were female, and sex was not considered as a biological variable in this study.

#### Cell lines

Breast cancer cell lines (MDA-MB-231, BT549, HCC1937, and MDA-MB-436) and HEK293T cells were obtained from American Type Culture Collection (ATCC). MDA-MB-231, MDA-MB-436, and HEK293T cells were cultured in DMEM (C11995500BT, Gibco) medium supplemented with 10% fetal bovine serum, 1% penicillin, and 1% streptomycin. BT549 and HCC1937 cells were cultured in RPMI-1640 (C11875500BT, Gibco) medium supplemented with 10% FBS, 1% penicillin, and 1% streptomycin. Cells were incubated in an incubator with 5% CO_2_ at 37°C. All cell lines were validated to be free of mycoplasma contamination.

### Method details

#### Plasmid construction, and transfection

For NAT10 knockout, silencing plasmids containing small hairpin RNA (shRNA) NAT10 were constructed based on the GV112. The sequence targeting NAT10 (sh-NAT10: 5′-GGAAUAUGGUGGACUAUCATT-3′) and a negative control shRNA (sh-NC: 5′-UUCUCCGAACGUGUCACGUTT-3') were synthesized by Genechem (Shanghai, China). The NAT10-overexpressing plasmid was synthesized by Genechem (Shanghai, China). All plasmids were verified through sequencing. Cells were seeded in six-well culture plates for plasmid or siRNA transfection. At a density of nearly 70%, cells were transfected using JetPrime (Polyplus, #114–15, Germany), following the manufacturer’s instructions. For lentiviral transduction, we used 4–6 μg/mL polybrene (Sigma-Aldrich, #107689, USA), and 1 μg/mL puromycin (Calbiochem, #540411, USA) to select transduced cells. To validate efficiency, stable knockout cell lines or overexpression cell lines were identified using quantitative reverse-transcription polymerase chain reaction (qRT-PCR) or western blotting. siRNA sequences were: siNAT10 1#: 5′-GGAAUAUGGUGGACUAUCATT-3′, siNAT10 2#: 5′-GUACUCCAAUAUCUUUGUUTT-3′, and siNAT10 3#: 5′-GAGUACUGUUGCACUCACATT-3′, siRAD51: 5′-GCAGUGAUGUCCUGGAUAATT-3’.

#### RNA isolation and qRT-PCR

Total RNA was extracted from cells using Trizol reagent (Invitrogen, #269201, USA). Then, 0.4 μg of RNA was reverse-transcribed into cDNA using a FastKing gDNA Dispelling RT SuperMix (TIANGEN, GKR118, China). Real-time PCR was conducted using FastStart Universal SYBR Green Master (ROX) (Roche, #04913914001, Switzerland) on a 7500 Fast Real-Time PCR system (Applied Biosystems, USA). Data were analyzed using the 2^–ΔΔCt^ method, and *ACTB* was regarded as the housekeeping gene. Primers used for qRT-PCR are listed in Table S1.

#### Western blotting

Cells and tissues were lysed using RIPA extraction reagent (Solarbio, R0010, China) supplemented with a protease inhibitor cocktail. Total protein concentration was measured by bicinchoninic acid analysis (Beyotime, P0010, China). Then, proteins were separated by 6–15% SDS PAGE, and transferred to 0.2 μm polyvinylidene fluoride membranes (Millipore, 03010040001, USA). After blocking with 5% skimmed milk for 90 min at room temperature, the membranes were incubated with a primary antibody at 4°C overnight. Blots were washed 3 × 10 min with TBST (containing 0.1% Tween 20). Next, blots were incubated with secondary antibodies conjugated to horseradish peroxidase (HRP) for 1 h at room temperature, and then washed with TBST 3 times, 10 min each. The blots were filmed by enhanced chemiluminescence (Meilunbio, MA0186, China) and developed using the BIO-RAD ChemiDoc XRS+ System. The following antibodies were used for Western blotting: NAT10 (Abcam, ab194297, 1:1000), β-actin (ZSGB-BIO, TA-09, 1:1000), BAX (Cell Signaling Technology, #2772, 1:1000), Bcl-2 (Wanleibio, WL01556, 1:500), γ-H2AX (Cell Signaling Technology, #9718, 1:1000), RAD51 (Abcam, ab133534, 1:1000), goat anti-mouse (Proteintech, No.SA00001-1, 1:10000), and goat anti-rabbit (Proteintech, No.SA00001-2, 1:10000).

#### ac4C dot blot

Samples of RNA were denatured in a heat block at 95°C for 3 min. Then, they were immediately placed on ice for 1 min and loaded onto Hybond-N+ membranes. Next, membranes were subjected to crosslinking using HL-2000 HybriLinker (UVP, USA) at UV254 nm. Thereafter, membranes were washed in 10 mL of TBST at room temperature (RT) for 5 min, blocked with 4% non-fat milk in TBST for 1 h at RT, and incubated with anti-ac4C antibody (Abcam, ab252215, 1:250) at 4°C overnight. The membranes were washed three times for 10 min in TBST. Then, the membranes were incubated with an HRP-conjugated secondary antibody at RT for 1 h and visualized using a chemiluminescent HRP substrate (Meilunbio, MA0186, China). Finally, the membranes were soaked with methylene blue for 15 min at RT and washed with TBST until the spots were clear. Images were captured as an internal reference.

#### Cell proliferation assays

2–3 × 10^3^ transfected cells were seeded in a 96-well plate at 37°C. After 24 h, cells were treated with the indicated concentrations of drugs for 24 h and 48 h. 10 μL CCK-8 solution was added to each well, and then plates were incubated at 37°C for 1 h. Cell proliferation curves were plotted by measuring the absorbance at 450 nm at each indicated time point.

#### Colony-formation assays

First, 1000 cells were plated in a six-well plate. After 24 h, cells were treated with the indicated concentrations of drugs for 48 h. Next, cells were cultured for two weeks in a complete medium containing 10% FBS. Cells were then fixed with 4% paraformaldehyde for 30 min and stained with crystal violet staining solution (Beyotime, C0121, China) for 30 min. The number of colonies was counted.

#### Apoptosis assay

First, 2 × 10^5^ cells were inoculated into a 6-well plate and treated with designated drugs for 48 h. The Annexin V-FITC Apoptosis Detection Kit (Beyotime, C1062, China) was used to analyze cell apoptosis according to the manufacturer’s instructions. Flow cytometry was conducted on a BD FACSverse flow cytometer.

#### Immunofluorescence staining

After specific treatment of cells, the Universal IF Toolkit was used for immunofluorescence staining following the manufacturer’s instructions. (Abbkine, KTD107, China). The primary antibody for immunofluorescence staining was γ-H2AX (Cell Signaling Technology, #9718, 1:200). The γ-H2AX foci were counted from at least 100 cells per sample.

#### Comet assays

After specific treatment, cells were subjected to comet assay using the Comet Assay Kit (KeyGen Bio-Tech, KGA1302, China) following the manufacturer’s instructions. Subsequently, images were captured using a fluorescence microscope (Nikon, Japan) and analyzed using casp1.2.3b2 software. For each group, at least 50 cells were analyzed. We calculated the degree of DNA damage as the percentage of tail DNA.

#### HR and non-homologous end-joining (NHEJ) reporter assays

Cells were transfected with pDR-GFP or pEJ5-GFP plasmids (Genechem, Shanghai, China) and stable DR-GFP and EJ5-GFP expressing cells were selected using puromycin screening. Then, cells were transfected with plasmids expressing I-Scel endonuclease (Genechem, Shanghai, China). After 48 h, cells were harvested and the proportion of GFP-positive cells was evaluated using flow cytometry. To investigate the role of remodelin in HR and NHEJ repair, cells were treated with 35 μM remodelin for 48 h and then transfected with I-Scel plasmid. After 48 h, cells were harvested and the proportion of GFP-positive cells was evaluated using flow cytometry.

#### Ac4c-rip

Total RNA was extracted using Trizol and ac4C-RIP (acRIP) was performed using EpiTM ac4C immunoprecipitation kit (Epibiotek, R1815, China). In brief, magnetic beads were incubated with either anti-ac4C antibody or IgG at 4°C for 6 h. Subsequently, the fragmented total RNA was incubated with the antibody-bead complex at 4°C overnight. Finally, ac4C-modified RNA was eluted and purified. qRT-PCR analysis was conducted on the recovered RNA. The primers for acRIP-qRT-PCR are shown in Table S1.

#### Molecular docking

The protein structure of NAT10 was docked with the mRNA structure of *RAD51* using HEX8.0 software (https://hex.loria.fr/). The top-scoring poses were sorted by affinity score and visualized using Pymol.

#### Animal experiments

Animal experiments were approved by the Medical Experimental Animal Care Committee of the Second Affiliated Hospital of Harbin Medical University and were conducted following the guidelines of the National Institutes of Health on animal care and ethical guidelines. We obtained 6–8 weeks female nude mice from Beijing Weitong Lihua Experimental Animal Technology Co., Ltd. Then, 5 × 10^6^ MDA-MB-231 or HCC1937 cells, either untransfected or transfected with sh-NC or sh-NAT10, were suspended in 200 μL serum-free medium and directly injected into the right breast fat pad. Olaparib (MCE, HY-10162) was administered at a dosage of 50 mg/kg by intraperitoneal injection 5 times per week. Remodelin (MCE, HY-16706A) was administered at a dosage of 3 mg/kg by intraperitoneal injection every four days. Mice weight was recorded every 3 days using a digital scale. Tumor volume was measured every 3 days using a caliper. The tumor volume was calculated as V = 1/2 (length x width^2^).

#### Immunohistochemistry

Tumor tissues were fixed, embedded, and sectioned (3 μm). Following the standard procedures, paraffin-embedded tissue sections were subjected to successive deparaffinization, antigen retrieval, background blocking, and target detection with the indicated antibodies. Detection was conducted using liquid DAB+ and counterstained with Carazzi’s hematoxylin. Gene expression was blindly measured by two pathologists. Briefly, the percentage of tumor cells with positive staining was as follows: 0 (no positive), 1 (positive ≤10%), 2 (10%< positive ≤25%), 3 (25%< positive ≤50%), 4 (50%< positive ≤75%), and 5 (positive >75%). The staining intensity was graded as follows: 0 (no staining), 1 (weak staining), 2 (moderate staining), and 3 (strong staining). The histochemistry score (H-score) was calculated by multiplying the proportion of positive tumor cells and the staining intensity. The following antibodies were used for immunohistochemistry (IHC): γ-H2AX (Cell Signaling Technology, #9718, 1:400), cleaved caspase-3 (Cell Signaling Technology, #9664, 1:400), Ki-67 (Cell Signaling Technology, #9449, 1:1000), and goat anti-rabbit (ZSGB-BIO, PV-6001).

### Quantification and statistical analysis

GraphPad Prism 10.1 was used to conduct statistical analyses. The Student’s t test was used to compare the experimental group with the control group. One-way ANOVA was used for multiple group comparisons. The results are expressed as mean ± the standard deviation (SD) of at least three independent experiments. ∗*p* < 0.05, ∗∗*p* < 0.01, ∗∗∗*p* < 0.001, and ∗∗∗∗*p* < 0.0001 were considered statistically significant.

## Supplemental information

Document S1. Figures S1–S11 and Table S1

Data S1. Uncropped western blot
