# Supplementary material for: Mechanistic study of N-acetyltransferase 10 deficiency enhancing olaparib sensitivity in triple negative breast cancer by inhibiting RAD51 N4-acetylcytidine modification
Source: iScience. 2025 Jun 9;28(7):112860. doi: 10.1016/j.isci.2025.112860 (PMC12221513; doi:10.1016/j.isci.2025.112860)
Supplement: Document S1. Figures S1–S11 and Table S1 [file mmc1.pdf]

iScience, Volume *28*

Supplemental information

Mechanistic study of N-acetyltransferase 10 deficiency enhancing olaparib sensitivity in triple negative breast cancer by inhibiting RAD51 N4-acetylcytidine modification

Hui Li, Hao Wu, Siwei Li, Qin Wang, Guozheng Li, Xin Ma, Yajie Gong, Yijun Chu, Shengye Jin, Xi Chen, Xianyu Zhang, and Da Pang

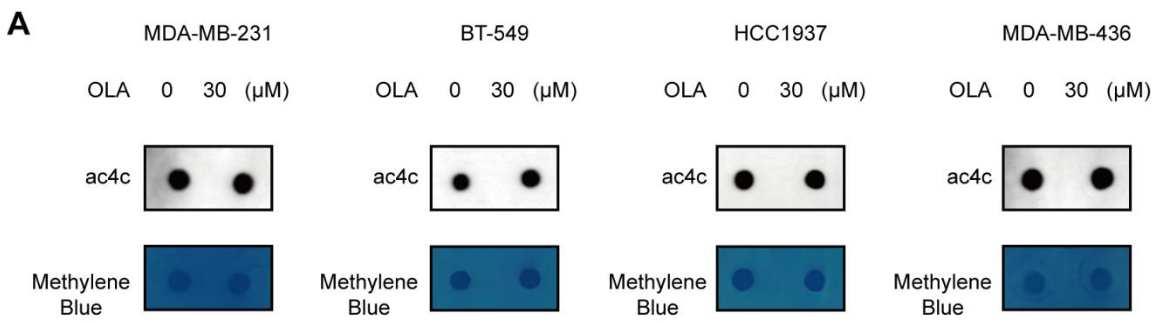

Figure S1. The level of ac4C modification after olaparib treatment in TNBC cells

(A) Dot blot assay to detect ac4C modification levels in TNBC cells treated with or without olaparib. ac4C, N4-acetylcytidine; TNBC, triple-negative breast cancer.

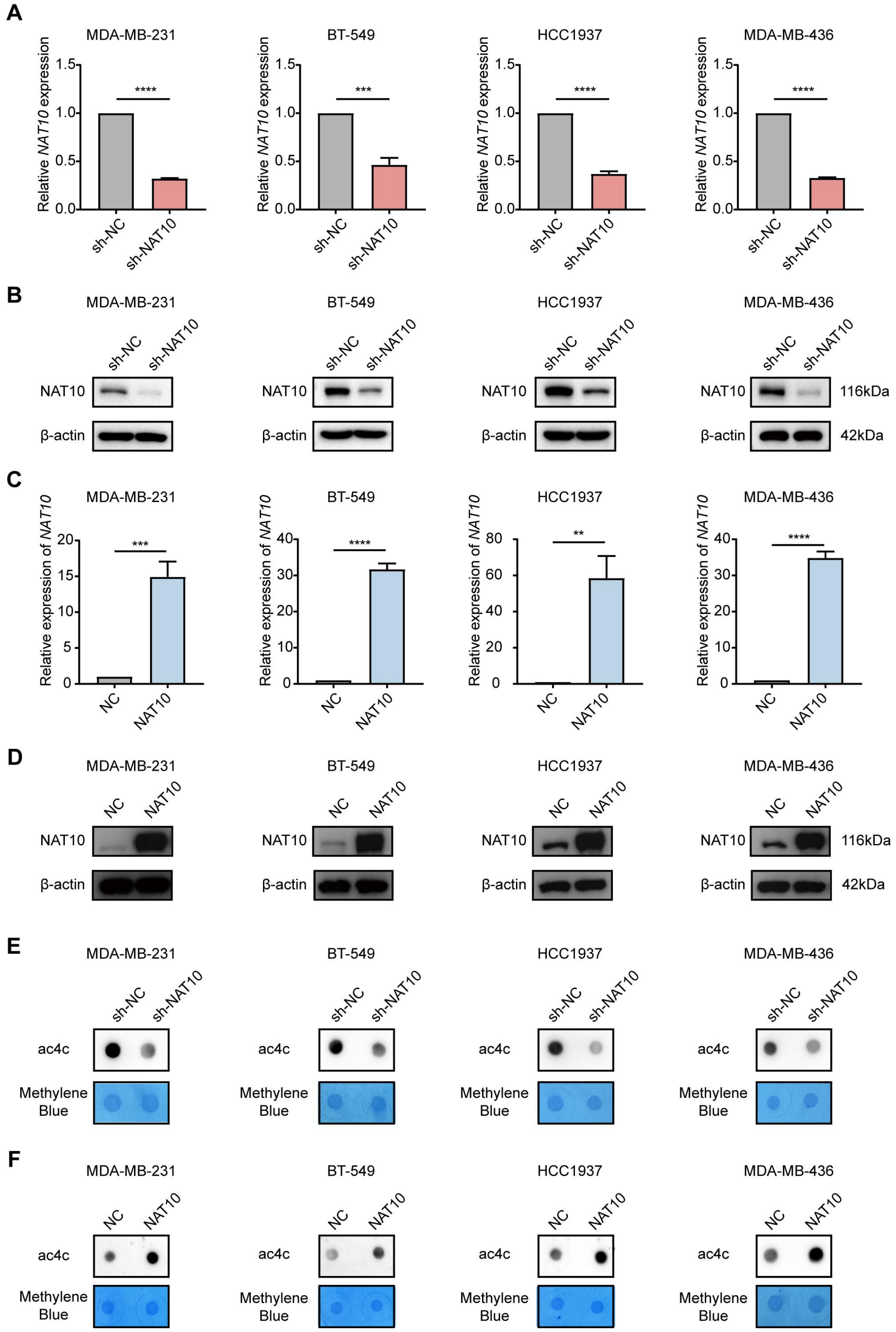

Figure S2. Generation of TNBC cell lines with NAT10 deletion and overexpression.

(A) qRT-PCR confirmed the knockout of *NAT10* by shRNA in MDA-MB-231, BT-549, HCC1937, and MDA-MB-436 cell lines. (B) Western blotting confirmed the knockout of NAT10 by shRNA in MDA-MB-231, BT-549, HCC1937, and MDA-MB-436 cell lines. (C) qRT-PCR confirmed the overexpression of *NAT10* in MDA-MB-231, BT-549, HCC1937, and MDA-MB-436 TNBC cell lines. (D) Western blotting confirmed the overexpression of NAT10 in MDA-MB-231, BT-549, HCC1937, and MDA-MB-436 cell lines. (E) Effect of NAT10 knockout on the expression of ac4C in MDA-MB-231, BT-549, HCC1937, and MDA-MB-436 cells detected by dot blotting. (F) Effect of NAT10 overexpression on ac4C expression in MDA-MB-231, BT-549, HCC1937, and MDA-MB-436 TNBC cells detected by dot blotting. Data are presented as the mean ± SD. ***P* < 0.01, ****P* < 0.001, and *****P* < 0.0001. TNBC, triple-negative breast cancer; NAT10, N-Acetyltransferase 10; ac4C, N4-acetylcytidine.

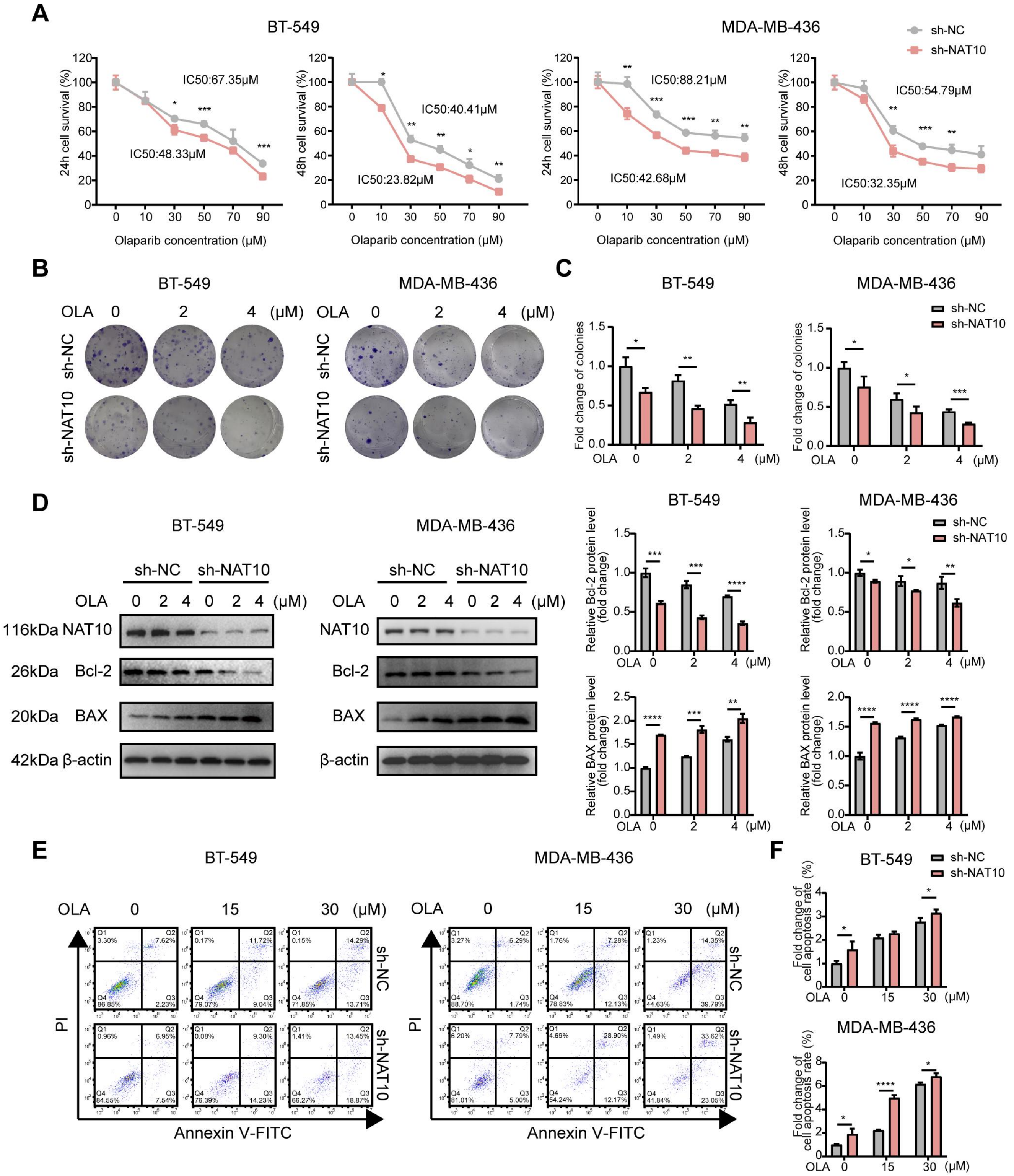

Figure S3. NAT10 knockout sensitized TNBC cells to olaparib.

(A) Cell survival analysis in BT-549 and MDA-MB-436 cells with sh-NC or sh-NAT10 treated with olaparib for 24 h and 48 h. (B and C) Representative images (B) and quantification (C) of colony formation assay conducted on BT-549 and MDA-MB-436 cells with sh-NC or sh-NAT10 after exposure to different concentrations of olaparib (0, 2, and 4 μM). (D) NAT10 knockout significantly enhanced the expression of the pro-apoptotic protein Bax and decreased the expression of the anti-apoptotic protein BCL2 in BT-549 and MDA-MB-436 cells treated with olaparib. Western blotting was used to quantify protein levels across various concentrations of olaparib (0, 2, and 4 μM) in BT-549 and MDA-MB-436 cell lines. (E and F) Flow cytometry analysis (E) and quantification (F) of apoptosis in BT-549 and MDA-MB-436 cells with sh-NC or sh-NAT10 after treatment with different concentrations of olaparib (0, 15, and 30 μM). Data are presented as means ± SD. *$P < 0.05$, **$P < 0.01$, ***$P < 0.001$, and ****$P < 0.0001$. NAT10, N-Acetyltransferase 10; TNBC, triple-negative breast cancer.

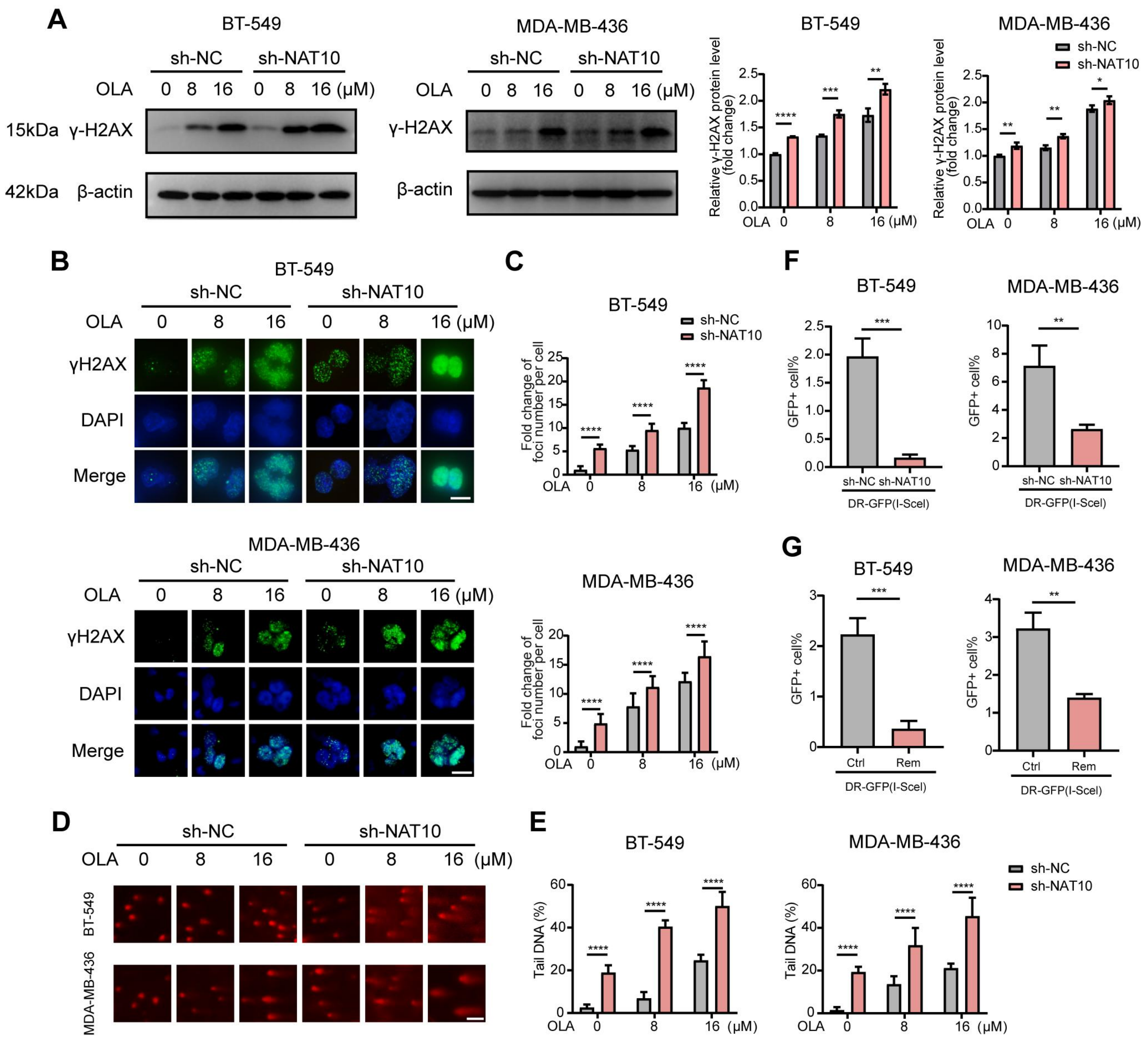

Figure S4. NAT10 suppression enhanced olaparib-induced DSBs.

(A) Western blotting illustrating the levels of γ-H2AX, a marker of DNA damage, in BT-549 and MDA-MB-436 cells with sh-NC or sh-NAT10 after treatment with different concentrations of olaparib (0, 8, and 16 μM). (B and C) Representative immunofluorescence images (B) and quantification (C) of γ-H2AX foci in BT-549 and MDA-MB-436 cells with sh-NC or sh-NAT10 treated with increasing concentrations of olaparib (0, 8, and 16 μM). γ-H2AX foci are stained green, and nuclei are counterstained with DAPI (blue). Merged images highlighting the colocalization of γ-H2AX foci within the nuclei. Scale bar: 20 μm. (D and E) Comet assay images (D) and quantification of tail DNA percentage (E) in BT-549 and MDA-MB-436 cells with sh-NC or sh-NAT10 after treatment with different concentrations of olaparib (0, 8, and 16 μM). Scale bar: 50 μm. (F) The frequency of GFP-positive (GFP+) cells of HR-mediated DSBs repair in sh-NC and sh-NAT10 BT-549 and MDA-MB-436 cells. (G) The frequency of GFP+ cells of HR-mediated DSBs repair in BT-549 and MDA-MB-436 cells treated with or without the NAT10 inhibitor remodelin. Data are presented as mean ± SD. *$P < 0.05$, **$P < 0.01$, ***$P < 0.001$ and ****$P < 0.0001$. NAT10, N-Acetyltransferase 10; DSBs, double strand breaks; HR, homologous recombination.

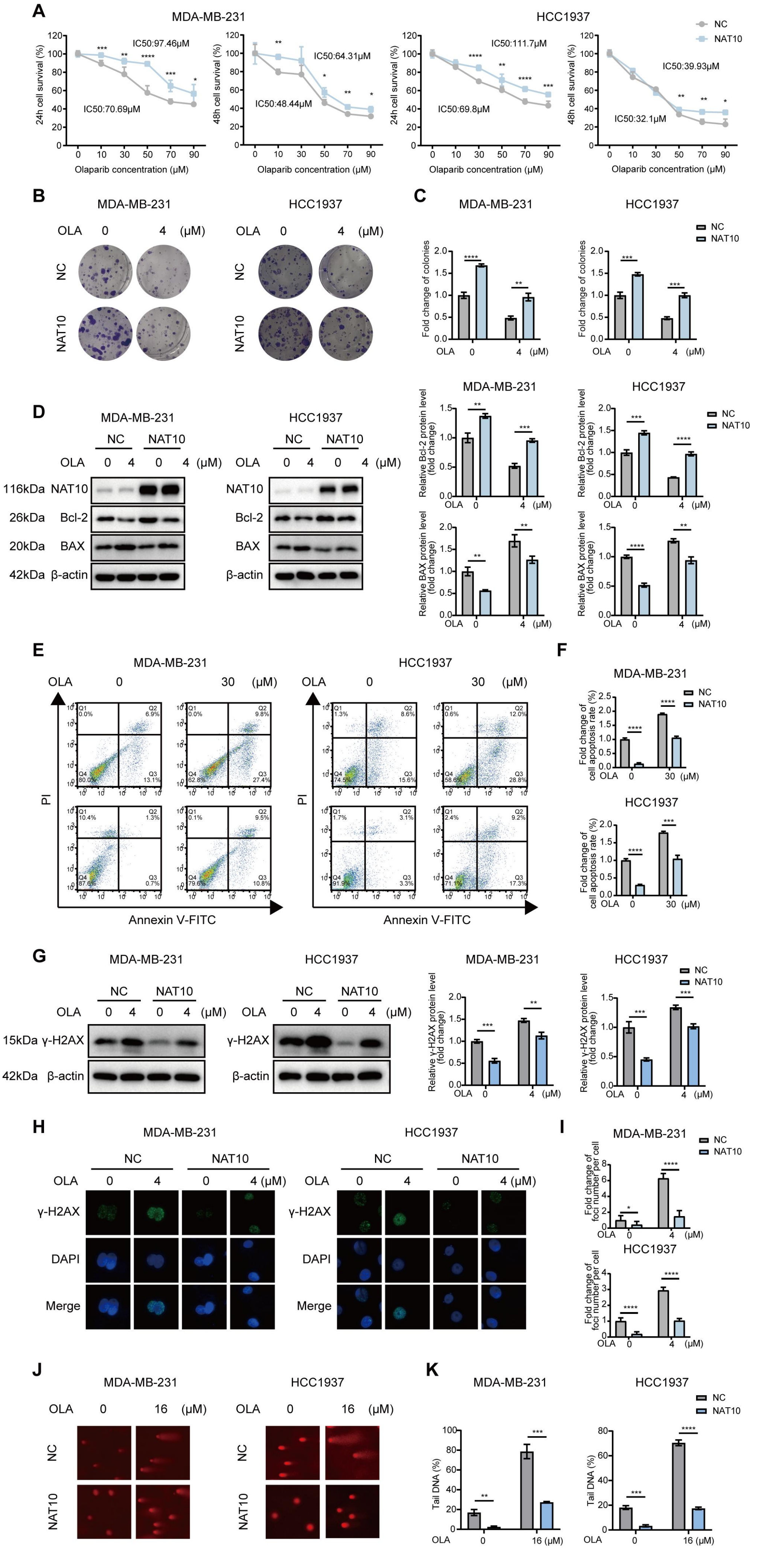
A
MDA-MB-231
HCC1937
NC
NAT10
24h cell survival (%)
48h cell survival (%)
Olaparib concentration (μM)
IC50:97.46μM
IC50:70.69μM
IC50:64.31μM
IC50:48.44μM
IC50:111.7μM
IC50:69.8μM
IC50:39.93μM
IC50:32.1μM
B
OLA 0 4 (μM)
C
Fold change of colonies
D
NC NAT10
OLA 0 4 0 4 (μM)
116kDa NAT10
26kDa Bcl-2
20kDa BAX
42kDa β-actin
Relative Bcl-2 protein level (fold change)
Relative BAX protein level (fold change)
E
OLA 0 30 (μM)
PI
Annexin V-FITC
F
Fold change of cell apoptosis rate (%)
G
15kDa γ-H2AX
42kDa β-actin
Relative γ-H2AX protein level (fold change)
H
γ-H2AX
DAPI
Merge
I
Fold change of foci number per cell
J
OLA 0 16 (μM)
K
Tail DNA (%)

Figure S5. NAT10 reduces olaparib sensitivity and inhibits olaparib-induced DSBs in TNBC cells

(A) Cell survival analysis in MDA-MB-231 and HCC1937 cells with control or NAT10-overexpression treated with olaparib for 24 h and 48 h. (B and C) Representative images (B) and quantification (C) of colony formation assay conducted on MDA-MB-231 and HCC1937 cells with control or NAT10-overexpression after exposure to different concentrations of olaparib (0 and 4 μM). (D) NAT10 significantly decreased the expression of the pro-apoptotic protein Bax and enhanced the expression of the anti-apoptotic protein BCL2 in MDA-MB-231 and HCC1937 cells treated with olaparib. (E and F) Flow cytometry analysis (E) and quantification (F) of apoptosis in MDA-MB-231 and HCC1937 cells with control or NAT10-overexpression after treatment with or without olaparib. (G) Western blotting illustrating the levels of γ-H2AX, a marker of DNA damage, in MDA-MB-231 and HCC1937 cells with control or NAT10-overexpression after treatment with or without olaparib. (H and I) Representative immunofluorescence images (H) and quantification (I) of γ-H2AX foci in MDA-MB-231 and HCC1937 cells with control or NAT10-overexpression treated with or without olaparib. γ-H2AX foci are stained green, and nuclei are counterstained with DAPI (blue). Merged images highlighting the colocalization of γ-H2AX foci within the nuclei. Scale bar: 20 μm. (J and K) Comet assay images (J) and quantification (K) of tail DNA percentage in MDA-MB-231 and HCC1937 cells with control or NAT10-overexpression after treatment with different concentrations of olaparib (0 and 16 μM). Scale bar: 50 μm. Data are presented as means ± SD. *P < 0.05, **P < 0.01, ***P < 0.001, and ****P < 0.0001. NAT10, N-Acetyltransferase 10; DSBs, double strand breaks; TNBC, triple-negative breast cancer.

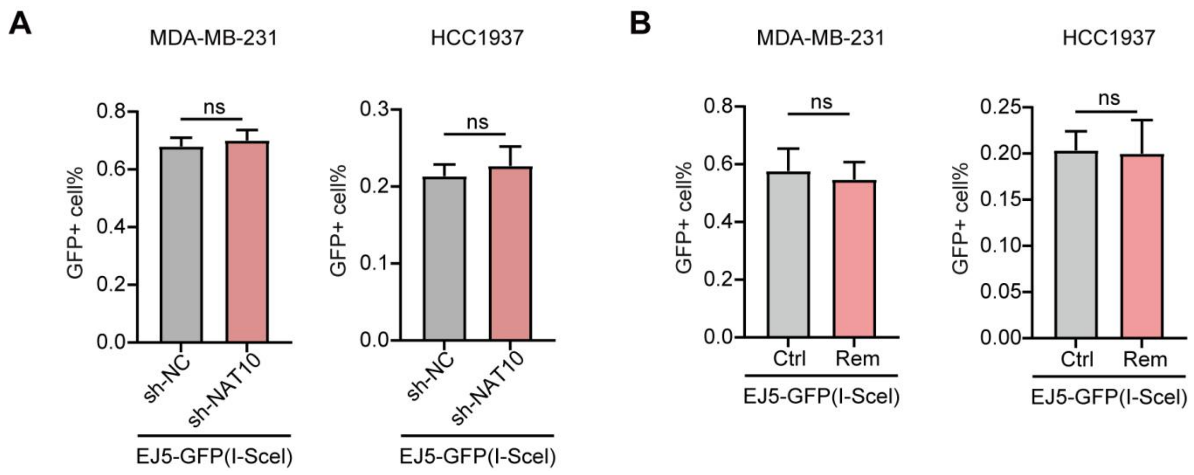

Figure S6. Assessment of NHEJ efficiency in response to NAT10 knockout or remodelin treatment in TNBC cells.

(A) The frequency of GFP-positive (GFP+) cells of NHEJ-mediated DSBs repair in MDA-MB-231 and HCC1937 cells with sh-NC or sh-NAT10. (B) The frequency of GFP+ cells of NHEJ-mediated DSBs repair in MDA-MB-231 and HCC1937 cells treated with or without the NAT10 inhibitor remodelin. Data are presented as mean ± SD. NHEJ, non-homologous end joining; NAT10, N-Acetyltransferase 10; TNBC, triple-negative breast cancer; DSBs, double strand breaks.

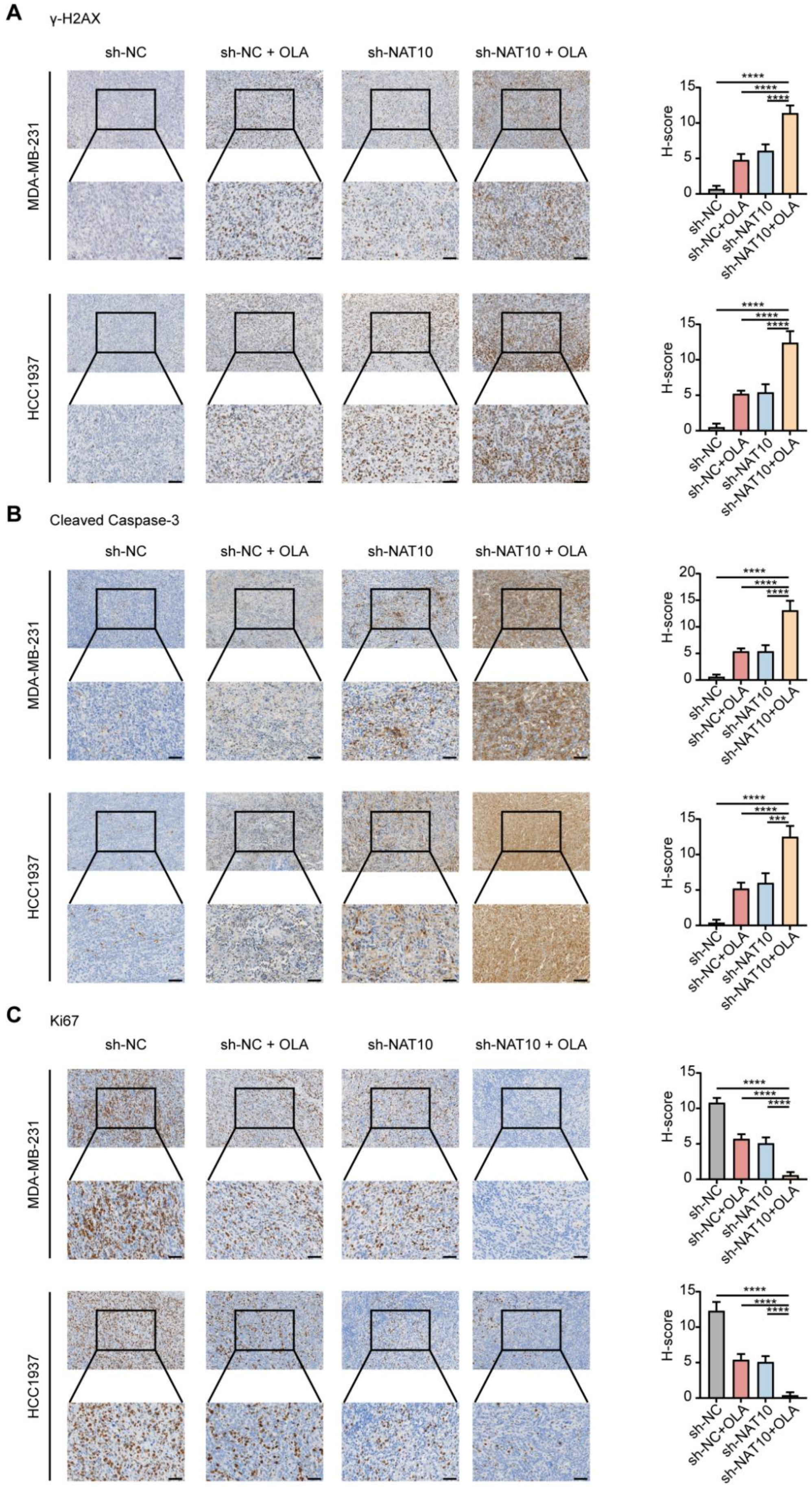

Figure S7. IHC analysis of γ-H2AX, cleaved caspase-3, and Ki67 expression in tumor tissues after olaparib treatment.

(A-C) Representative images and quantification of γ-H2AX (A), cleaved caspase 3 (B), and Ki67 (C) expression in the tumor by IHC. Scale bar: 50 μm. Data are presented as mean ± SD. ***P < 0.001 and ****P < 0.0001. IHC, immunohistochemistry.

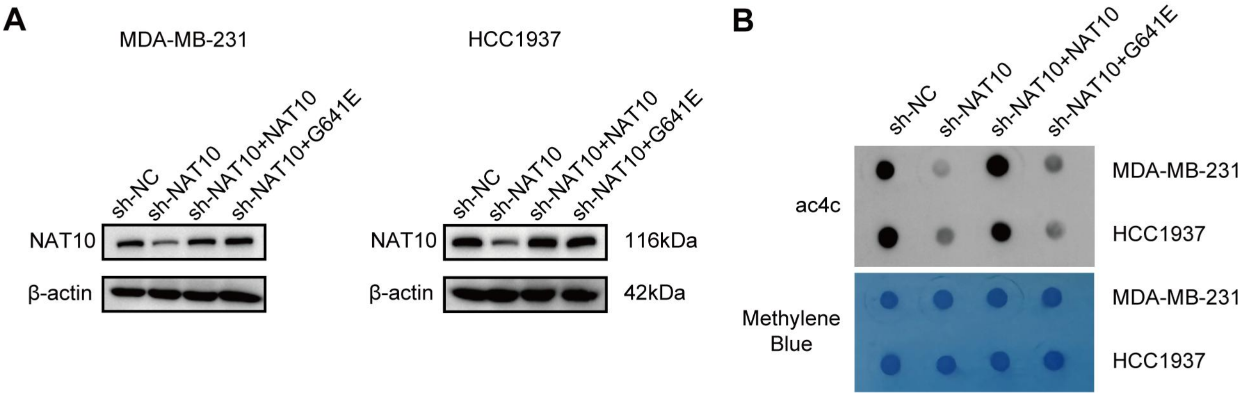

Figure S8. Generation of TNBC cell lines with NAT10 deletion and rescue.

(A) Western blot analysis was performed to confirm the level of NAT10 in sh-NC, sh-NAT10, sh-NAT10 + wild-type NAT10, and sh-NAT10 + G641E mutant NAT10 TNBC cells. (B) Dot blot assay to detect ac4C modification levels in the above cells. TNBC, triple-negative breast cancer; NAT10, N-Acetyltransferase 10; ac4C, N4-acetylcytidine.

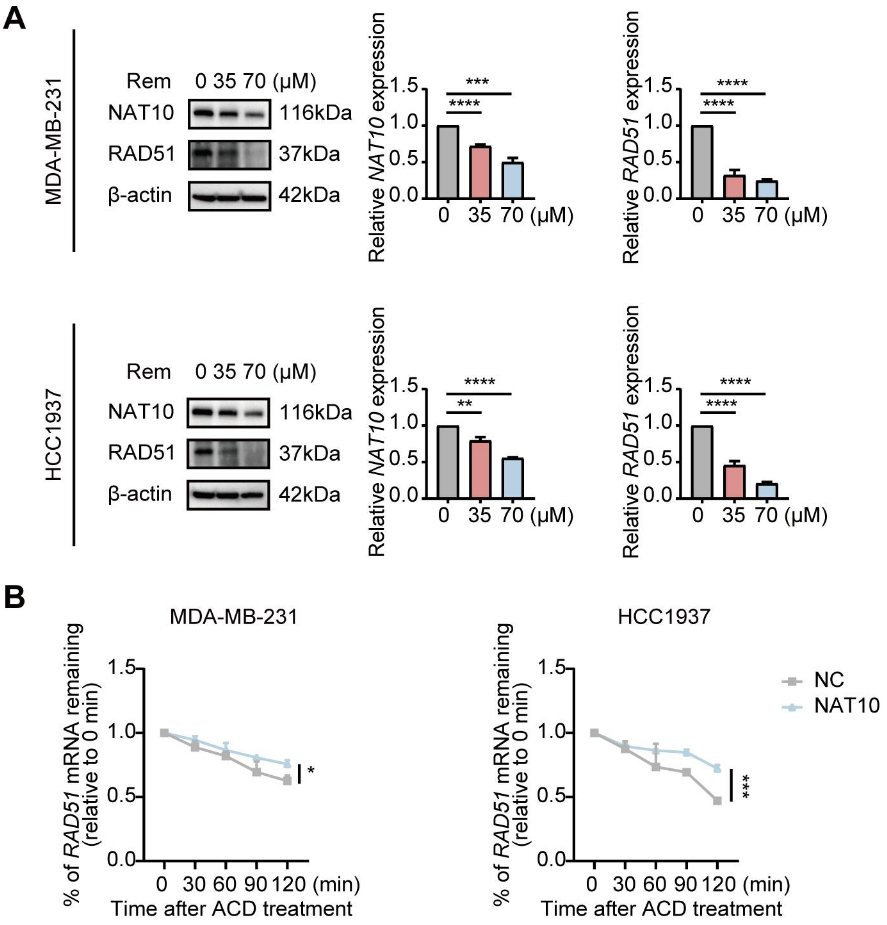

Figure S9. Effects of remodelin on RAD51 expression and of NAT10 overexpression on *RAD51* mRNA stability. (A) Western blotting and qRT-PCR for the expression levels of NAT10 and RAD51 in MDA-MB-231 and HCC1937 cells with remodelin treatment. (B) mRNA stability assay to evaluate *RAD51* mRNA degradation over time in control and NAT10-overexpressing cells treated with ACD. Data are presented as mean ± SD. $^*P < 0.05$, $^{**}P < 0.01$, $^{***}P < 0.001$, and $^{****}P < 0.0001$. NAT10, N-Acetyltransferase 10; ACD, actinomycin D.

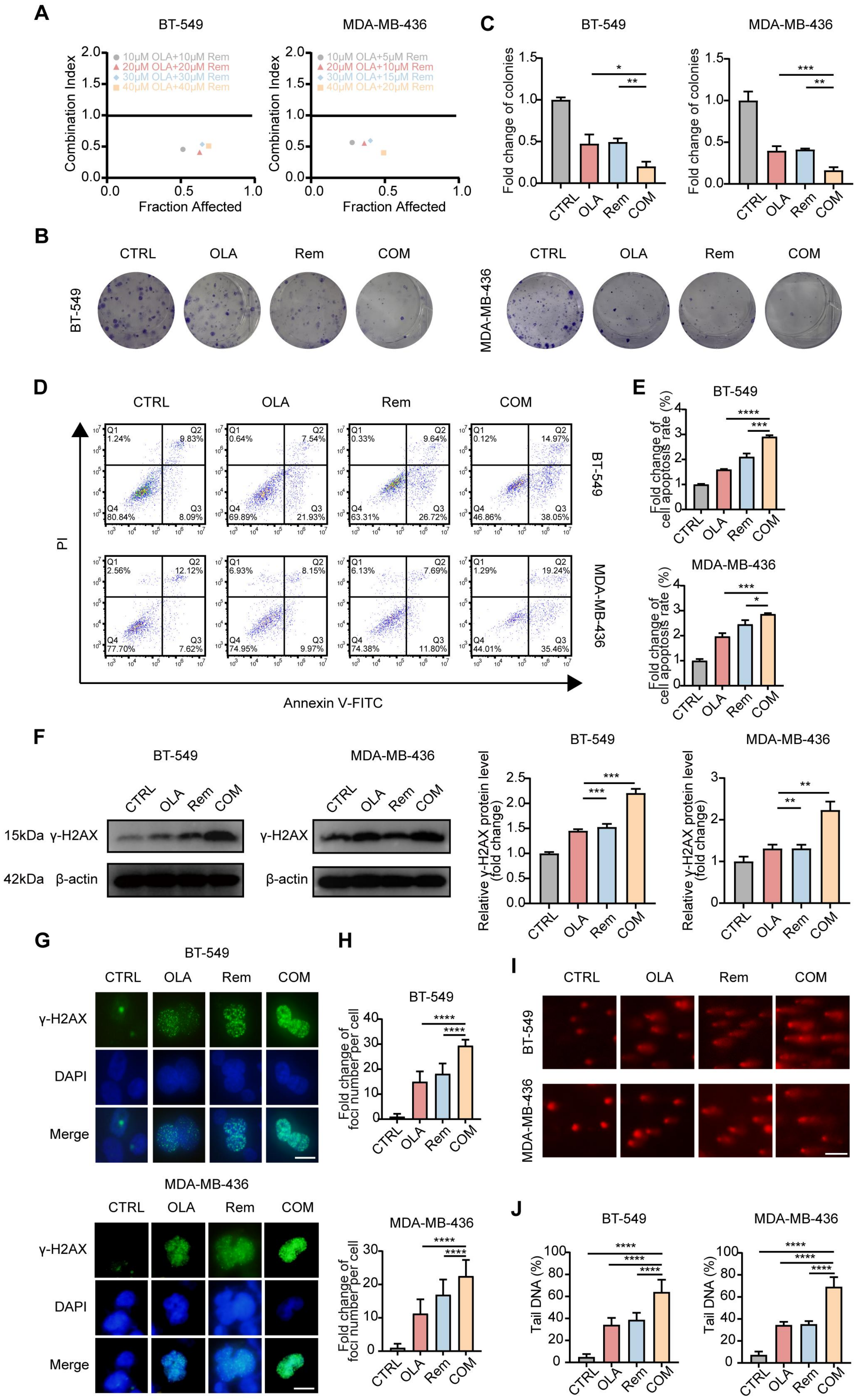
A
BT-549
MDA-MB-436
Combination Index
Fraction Affected
10μM OLA+10μM Rem
20μM OLA+20μM Rem
30μM OLA+30μM Rem
40μM OLA+40μM Rem
10μM OLA+5μM Rem
20μM OLA+10μM Rem
30μM OLA+15μM Rem
40μM OLA+20μM Rem
B
C
Fold change of colonies
D
E
PI
Annexin V-FITC
Fold change of cell apoptosis rate (%)
Q1
Q2
Q3
Q4
1.24%
9.83%
8.09%
80.84%
0.64%
7.54%
21.93%
69.89%
0.33%
9.64%
26.72%
63.31%
0.12%
14.97%
38.05%
46.86%
2.56%
12.12%
7.62%
77.70%
6.93%
8.15%
9.97%
74.95%
6.13%
7.69%
11.80%
74.38%
1.29%
19.24%
35.46%
44.01%
F
15kDa γ-H2AX
42kDa β-actin
Relative γ-H2AX protein level (fold change)
G
H
γ-H2AX
DAPI
Merge
Fold change of foci number per cell
I
J
Tail DNA (%)
CTRL
OLA
Rem
COM

Figure S10. Remodelin enhanced the sensitivity to olaparib and enhanced olaparib-induced DSBs in TNBC cells.

(A) Scatter plots representing the CI for BT-549 and MDA-MB-436 cell lines treated with the combination of olaparib and remodelin. The CI values are plotted against the fraction affected, indicating the level of drug synergy, where a CI value less than 1 suggested synergism. (B and C) Representative images (B) and quantification (C) of colony formation assay in BT-549 and MDA-MB-436 cells treated with DMSO (CTRL), olaparib (2 μM), remodelin (35 μM), or the combination of olaparib (2 μM) and remodelin (35 μM). (D and E) Flow cytometry analysis (D) and quantification (E) of apoptosis in BT-549 and MDA-MB-436 cells treated with DMSO (CTRL), olaparib (2 μM), remodelin (35 μM), or the combination of olaparib (2 μM) and remodelin (35 μM). (F) Western blot analysis was performed to determine the levels of γ-H2AX in BT-549 and MDA-MB-436 cell lines treated with DMSO (CTRL), olaparib (2 μM), remodelin (35 μM), or a combination of olaparib (2 μM) and remodelin (35 μM). (G and H) Representative images (G) and quantification (H) of γ-H2AX foci formation in the above cells. Scale bar: 20 μm. (I and J) Comet assay images (I) and quantification of tail DNA percentage (J) in the above cells. Scale bar: 50 μm. Data are presented as mean ± SD. **P* < 0.05, ***P* < 0.01, ****P* < 0.001 and *****P* < 0.0001. DSBs, double strand breaks; TNBC, triple-negative breast cancer; CI, combination index.

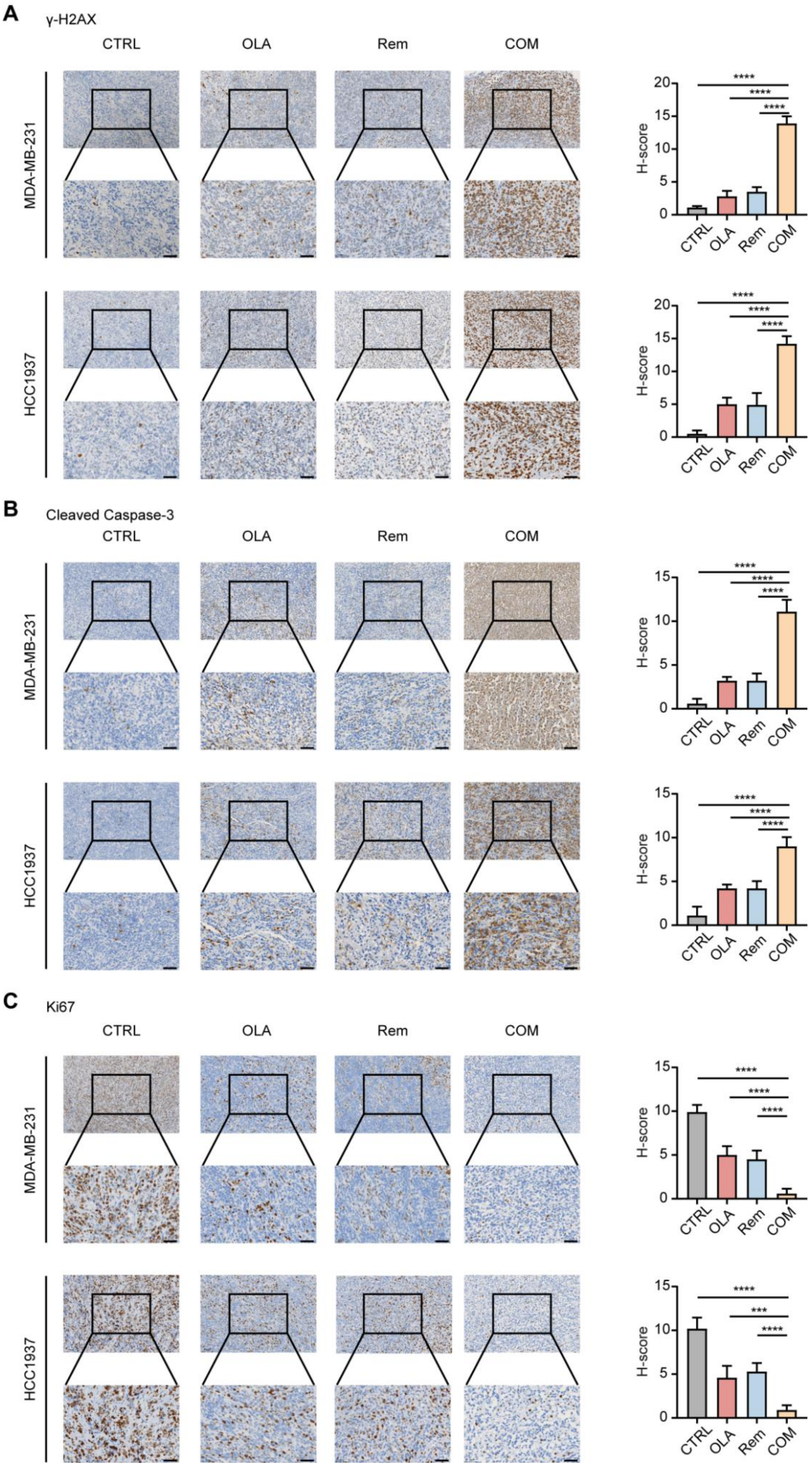

Figure S11. Co-administration of remodelin and olaparib increased γ-H2AX and cleaved caspase 3 levels but decreased Ki67 in MDA-MB-231 and HCC1937 tumor xenografts.

(A-C) Representative images and quantification of γ-H2AX (A), cleaved caspase 3 (B), and Ki67 (C) expression in the tumor by IHC. Scale bar: 50 μm. Data are presented as mean ± SD. ***P < 0.001 and ****P < 0.0001. IHC, immunohistochemistry.

Table S1. Human primers used in this study

**Gene**	**primers (5'–3')**
NAT10-F	GGAATATGGTGGACTATCA
NAT10-R	GTACTCCAATATCTTTGTT
RAD51-F	CAACCCATTTCACGGTTAGAGC
RAD51-R	TTCTTTGGCGCATAGGCAACA
RAD52-F	CCAGAAGGTGTGCTACATTGAG
RAD52-R	ACAGACTCCCACGTAGAACTTG
BRCA1-F	GAAACCGTGCCAAAAGACTTC
BRCA1-R	CCAAGGTTAGAGAGTTGGACAC
BRCA2-F	CACCCACCCTTAGTTCTACTGT
BRCA2-R	CCAATGTGGTCTTTGCAGCTAT
TP53BP1-F	ATGGACCCTACTGGAAGTCAG
TP53BP1-R	TTTCTTTGTGCGTCTGGAGATT
RBBP8-F	CAGGAACGAATCTTAGATGCACA
RBBP8-R	GCCTGCTCTTAACCGATCTTCT
PALB2-F	AGGATCTCTCACCGCAGCTAA
PALB2-R	TCAGGCCCAACATCAAGTGTG
ATM-F	ATCTGCTGCCGTCAACTAGAA
ATM-R	GATCTCGAATCAGGCGCTTAAA
RPA1-F	GGGGATACAAACATAAAGCCCA
RPA1-R	CGATAACGCGGCGGACTATT
MRE11-F	ATGCAGTCAGAGGAAATGATACG
MRE11-R	CAGGCCGATCACCCATACAAT
ACTB-F	GGGAAATCGTGCGTGACATT
ACTB-R	GGAACCGCTCATTGCCAAT
RAD51-F(ac4C)	CCAGGCTGGAGTGCAATAGC
RAD51-R(ac4C)	GGCTGAGGCAGGAGAATCAC
