# Supplementary material for: Mechanistic study of N-acetyltransferase 10 deficiency enhancing olaparib sensitivity in triple negative breast cancer by inhibiting RAD51 N4-acetylcytidine modification
Source: iScience. 2025 Jun 9;28(7):112860. doi: 10.1016/j.isci.2025.112860 (PMC12221513; doi:10.1016/j.isci.2025.112860)

**Uncropped western blot**

Figure 1D  left panel

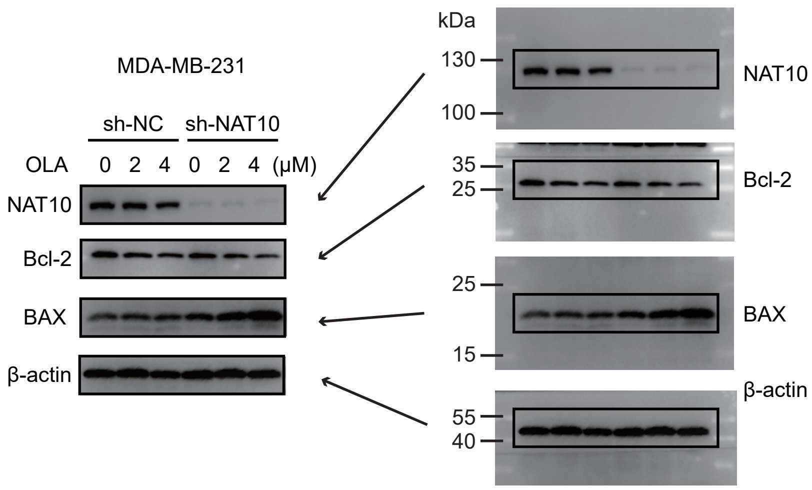


Figure 1D  right panel

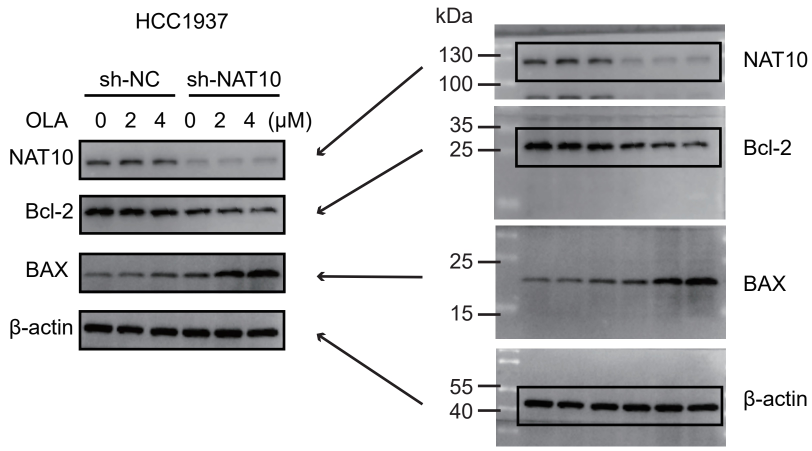


Figure 2B  upper panel

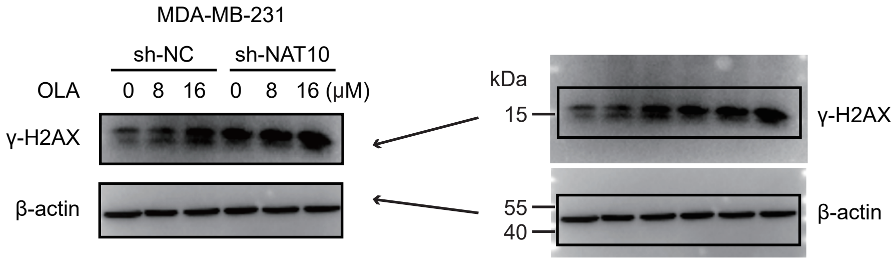


Figure 2B  lower panel

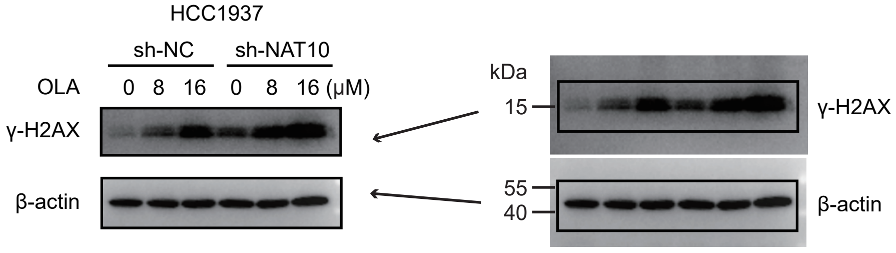

## Figure 3F upper panel

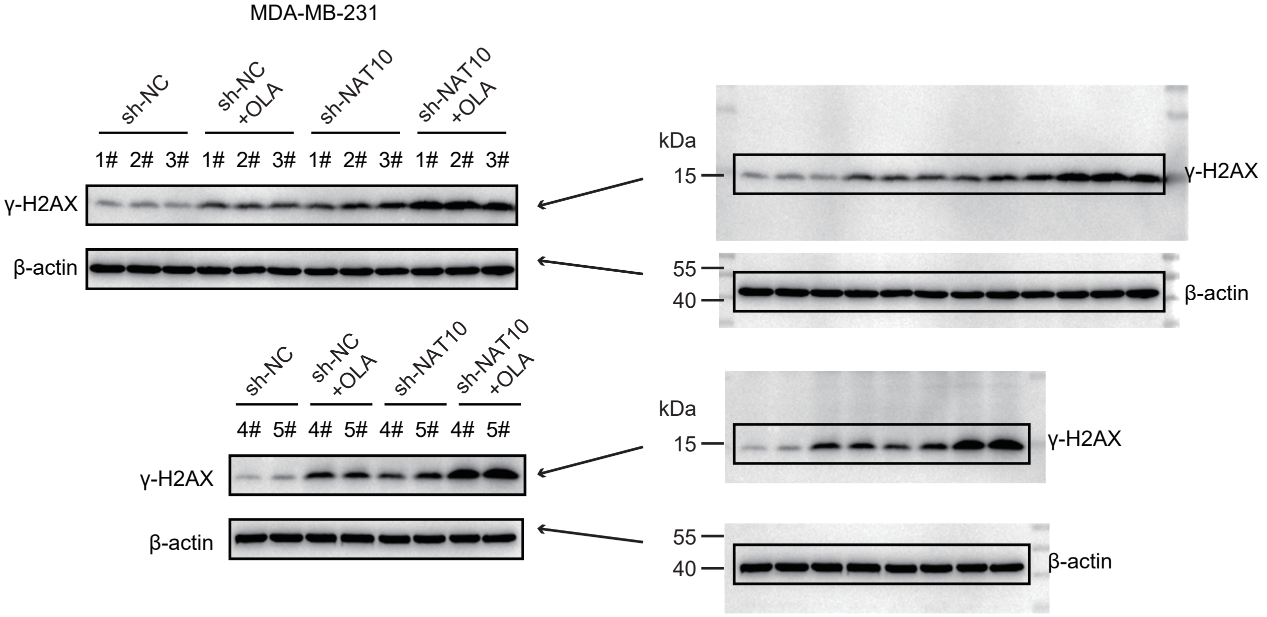


## Figure 3F lower panel

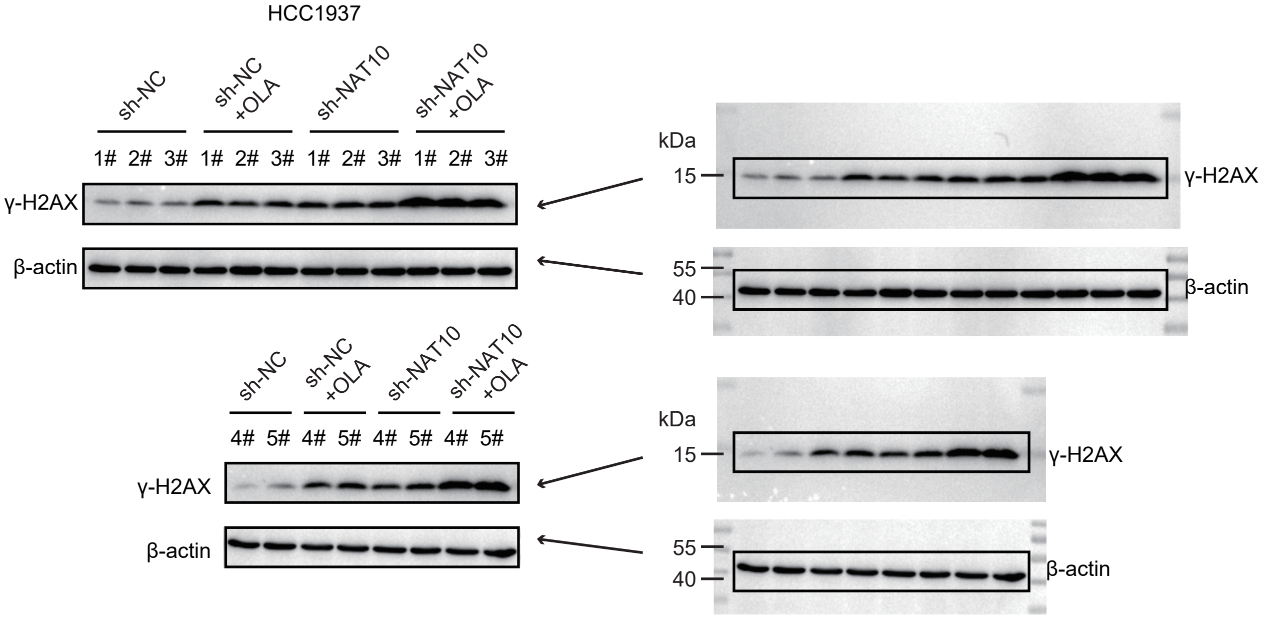


## Figure 4D left panel

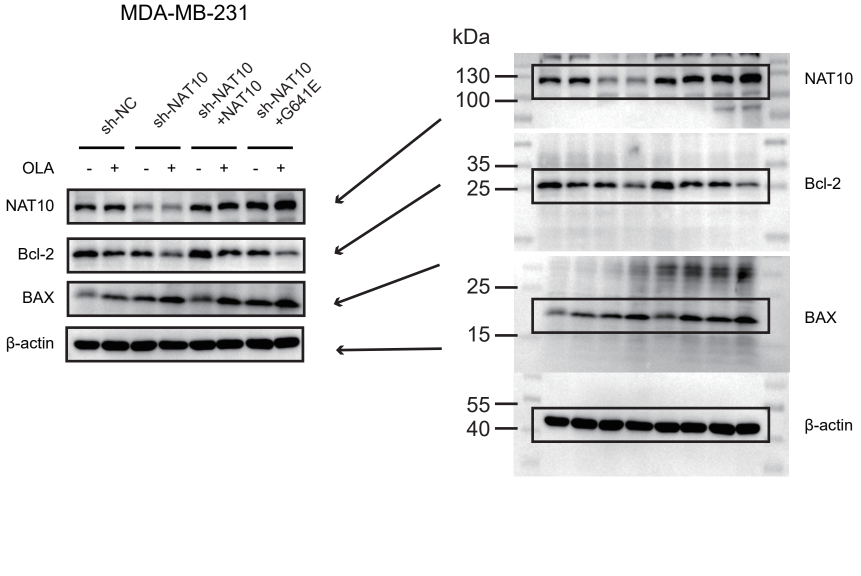

## Figure 4D right panel

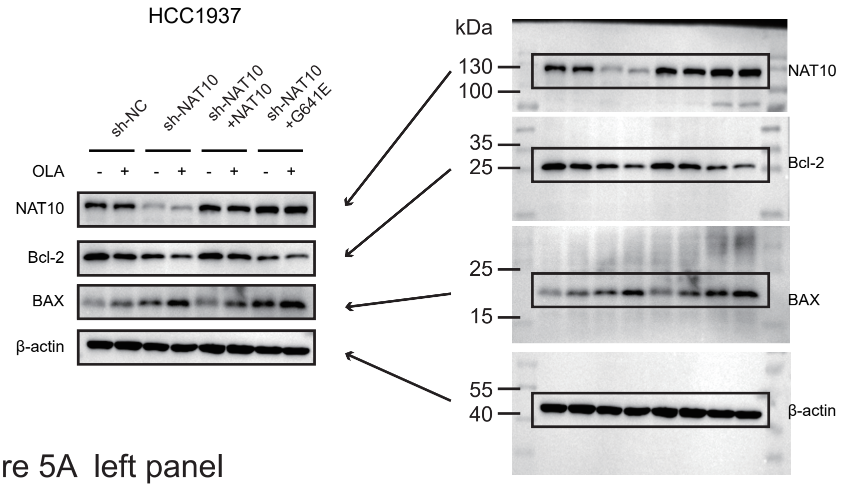


## Figure 5A left panel

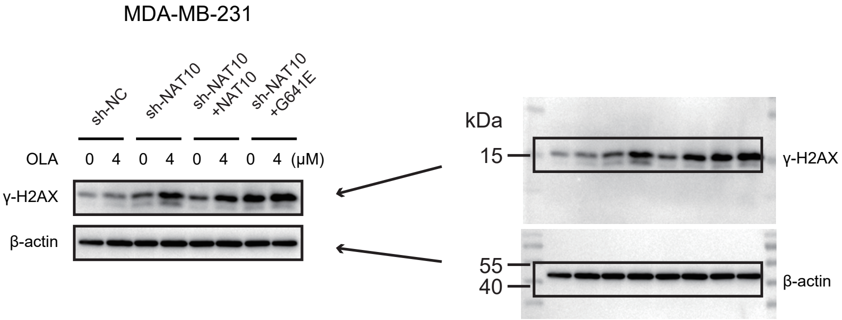


## Figure 5A right panel

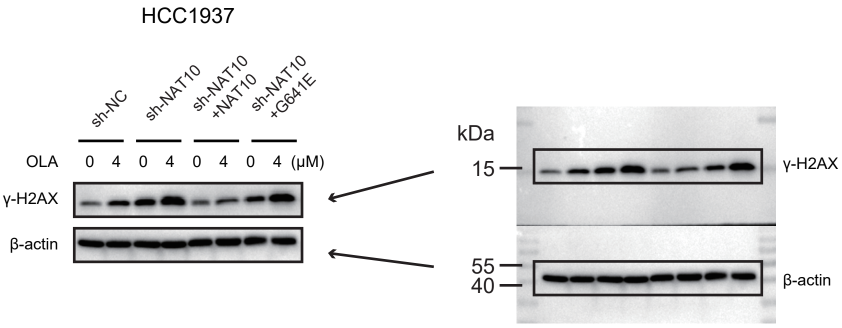


## Figure 6D upper panel

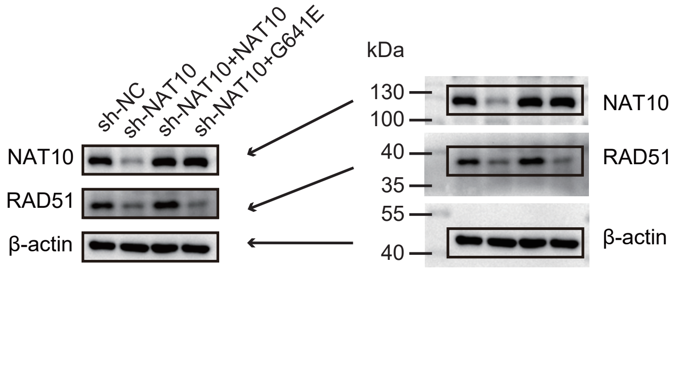


## Figure 6D lower panel

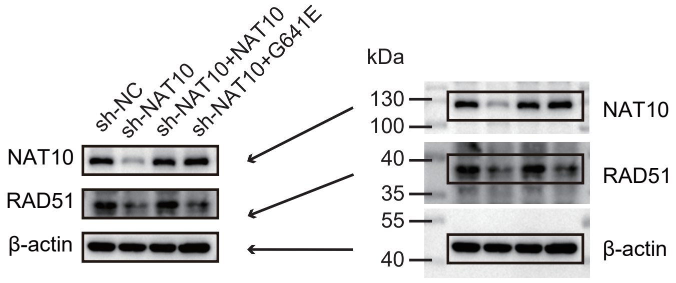

## Figure 7E left panel

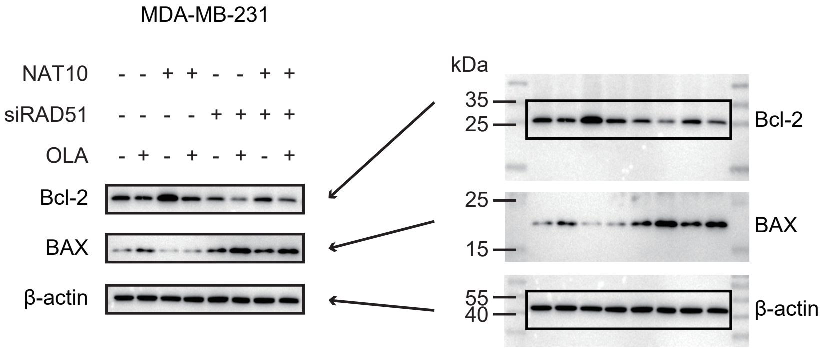


## Figure 7E right panel

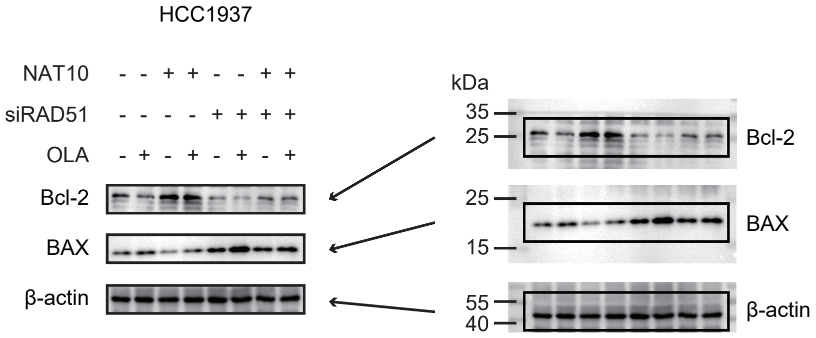


## Figure 8A left panel

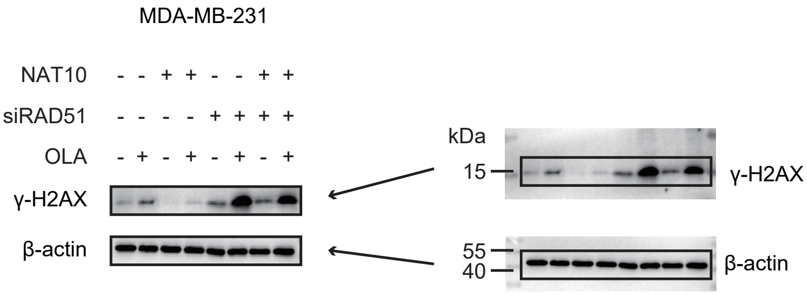


## Figure 8A right panel

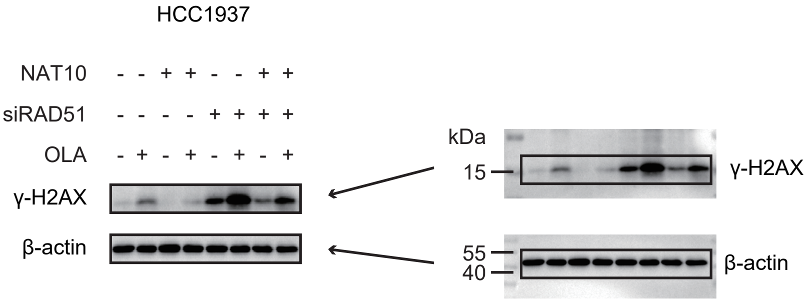

## Figure 9F left panel

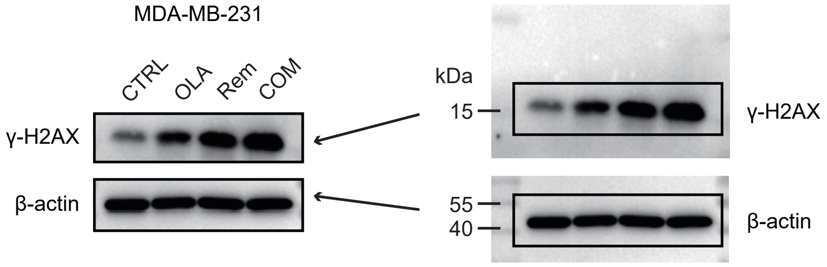


## Figure 9F right panel

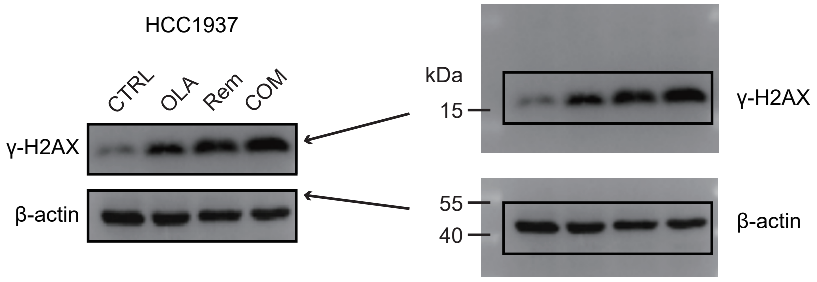


## Figure 10F upper panel

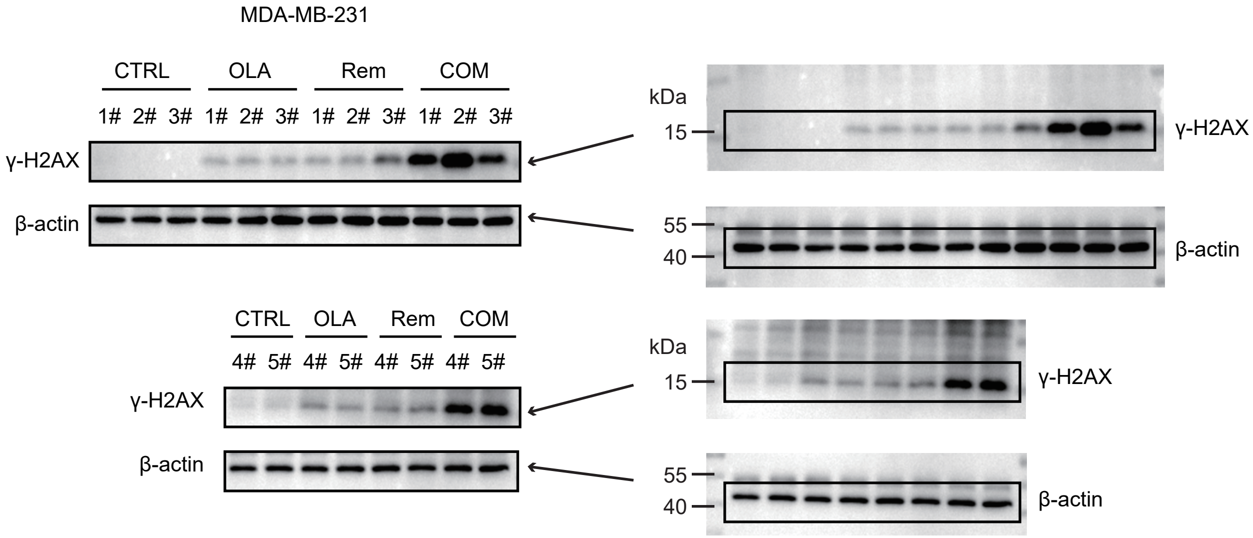


## Figure 10F lower panel

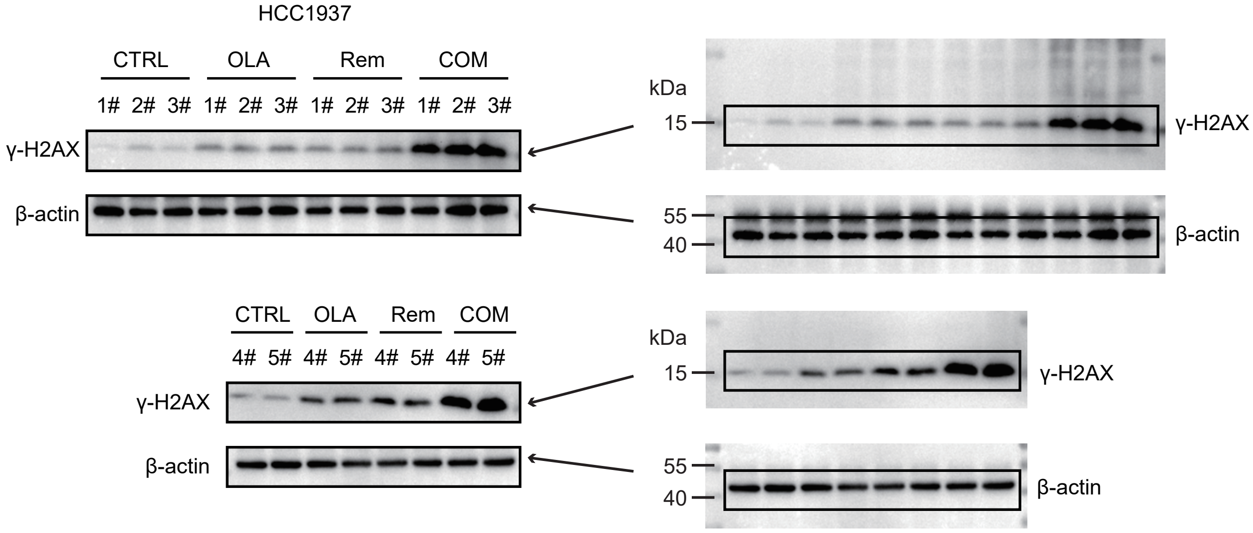

Figure S2B first panel

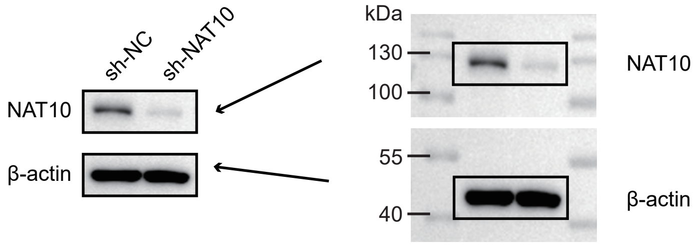


Figure S2B second panel

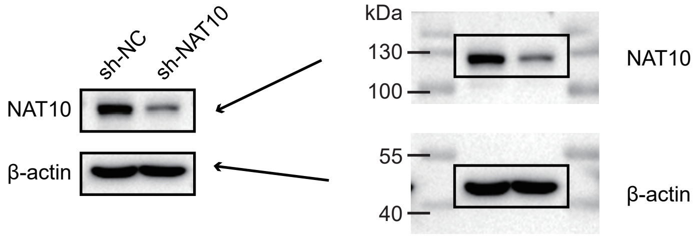


Figure S2B third panel

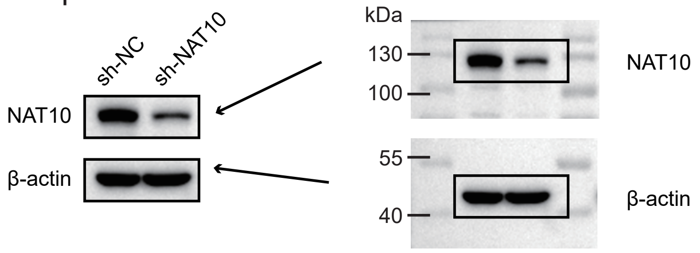


Figure S2B fourth panel

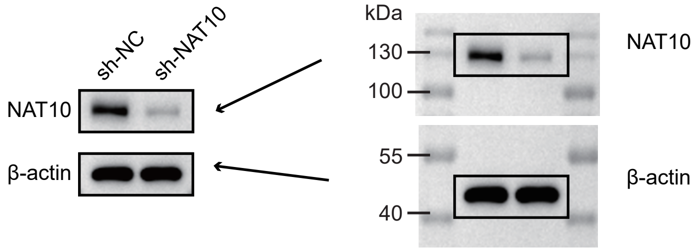


Figure S2D first panel

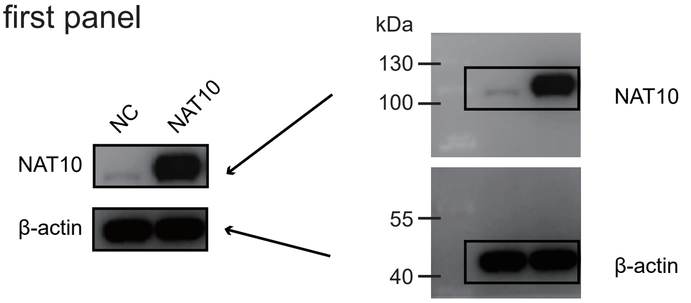


Figure S2D second panel

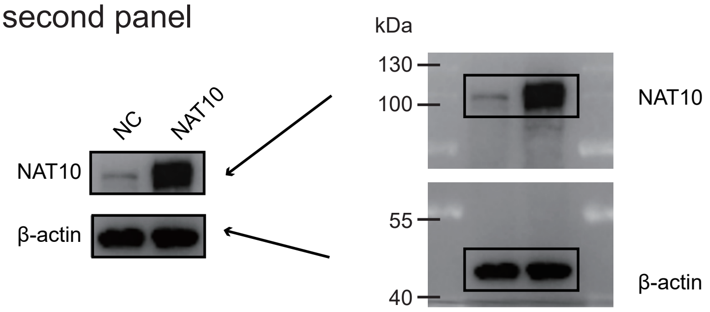


Figure S2D third panel

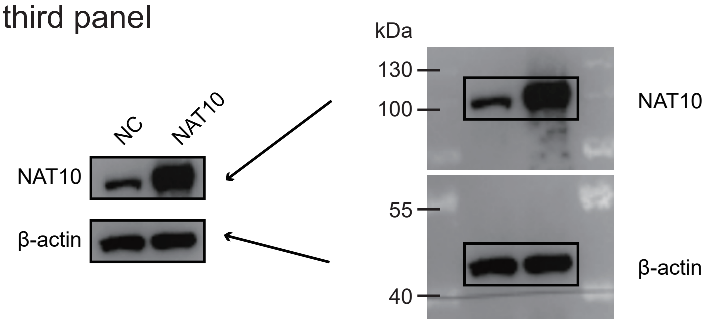

## Figure S2D fourth panel

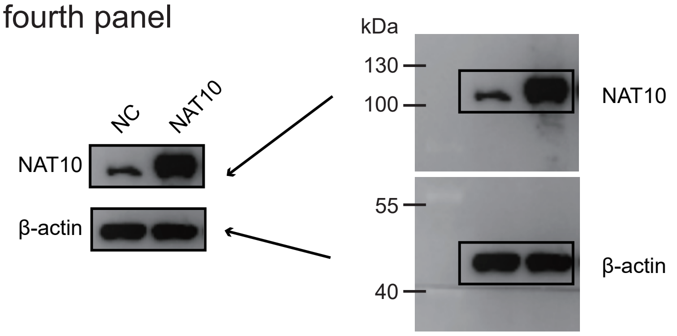


## Figure S3D left panel

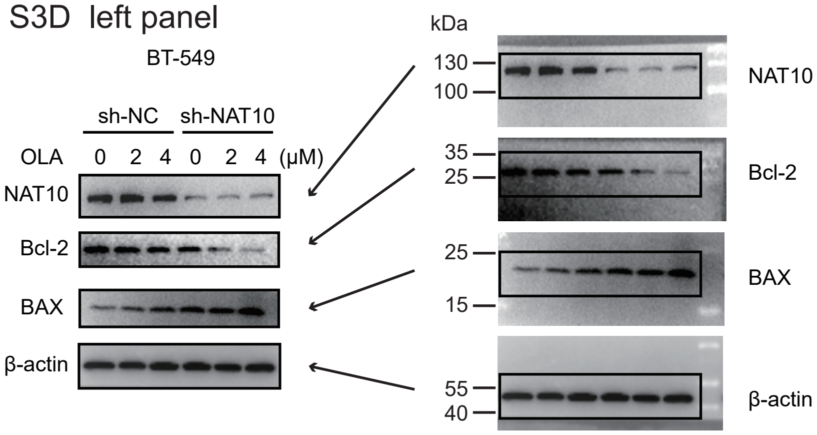


## Figure S3D right panel

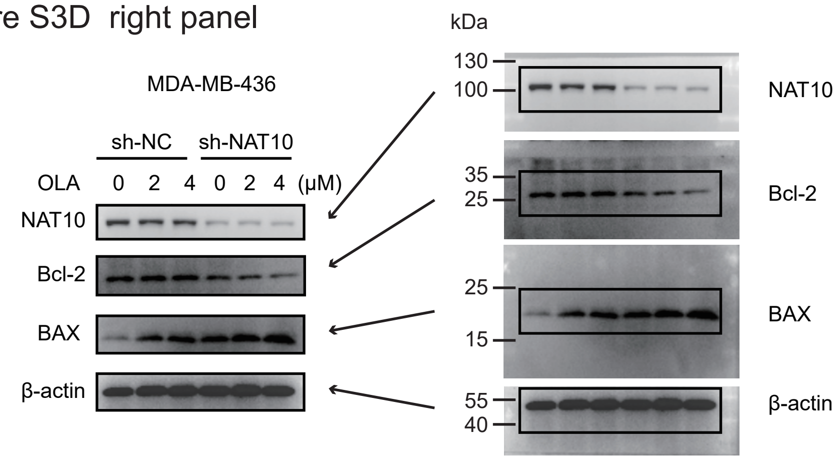


## Figure S4A left panel

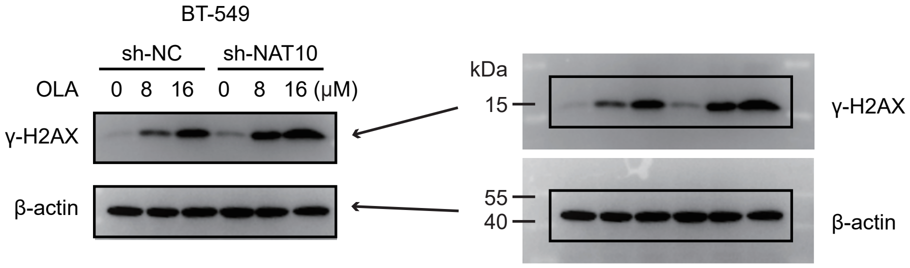


## Figure S4A right panel

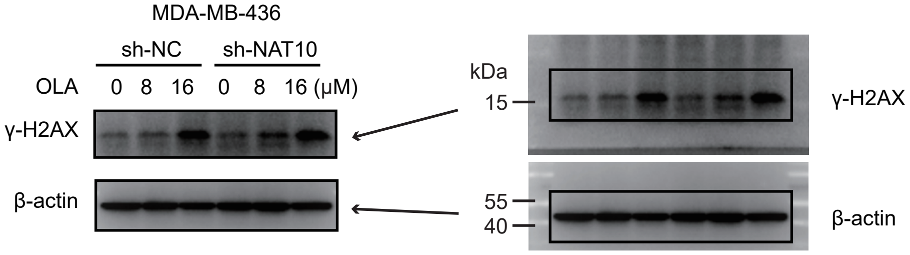

## Figure S5D left panel

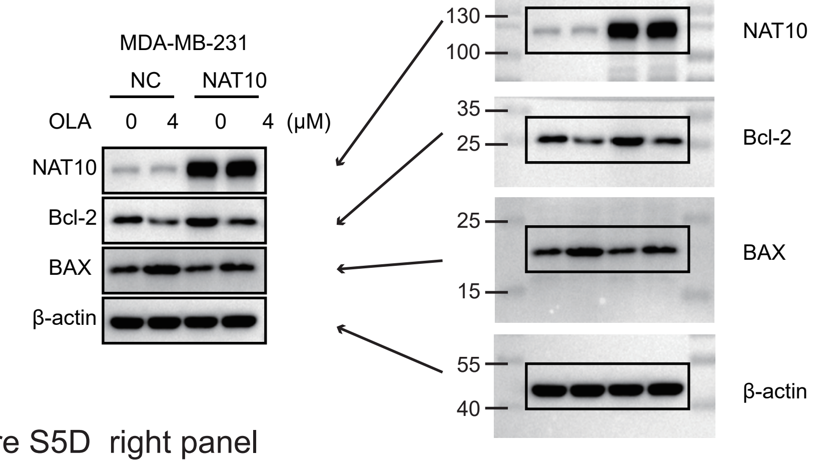


## Figure S5D right panel

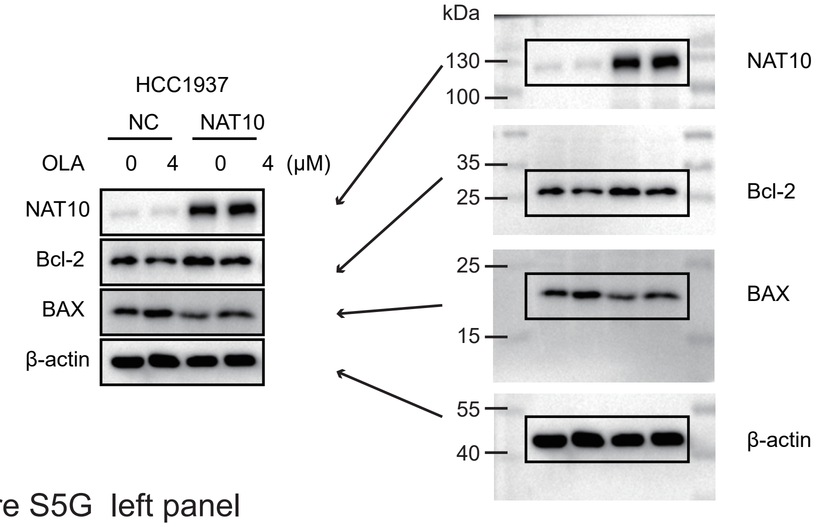


## Figure S5G left panel

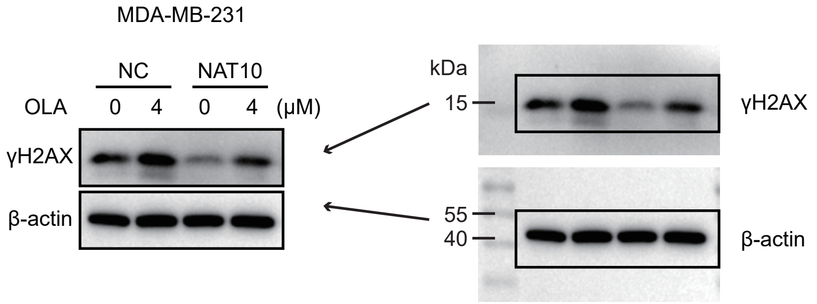


## Figure S5G right panel

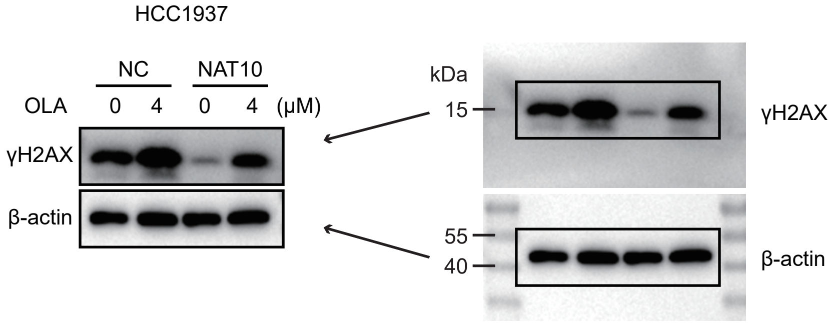


## Figure S8A left panel

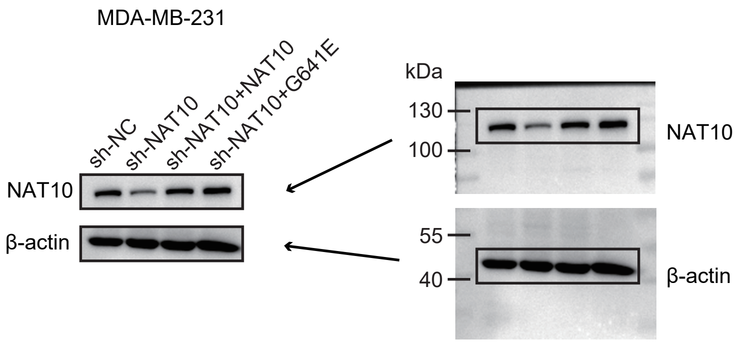

## Figure S8 right panel

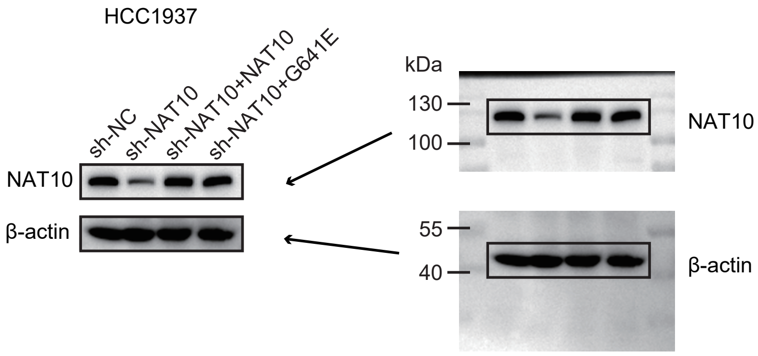


## Figure S9 upper panel

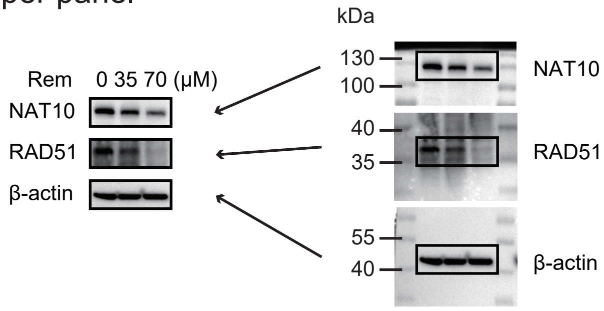


## Figure S9 lower panel

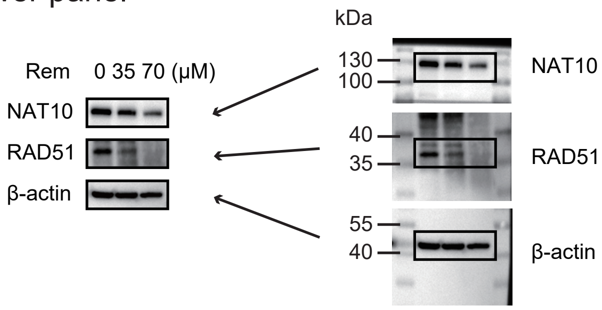


## Figure S10 left panel

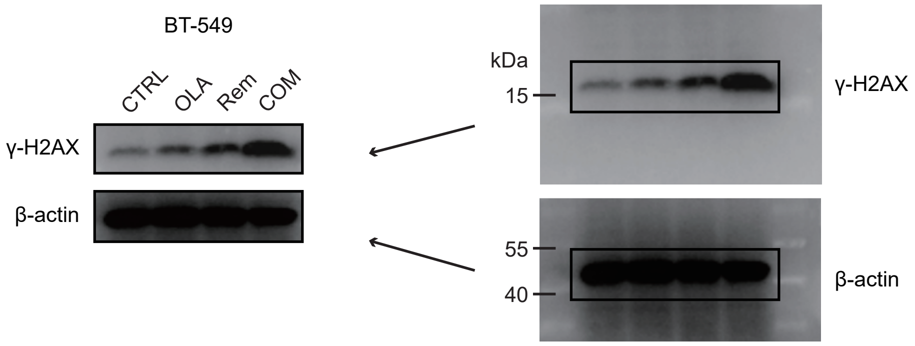


## Figure S10 right panel

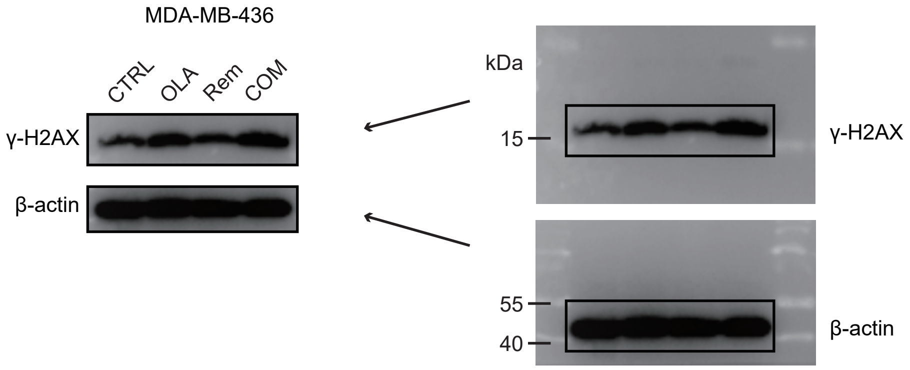

Supplement: Data S1. Uncropped western blot [file mmc2.pdf]
